# Use of Chitosan as Copper Binder in the Continuous Electrochemical Reduction of CO_2_ to Ethylene in Alkaline Medium

**DOI:** 10.3390/membranes12080783

**Published:** 2022-08-15

**Authors:** Aitor Marcos-Madrazo, Clara Casado-Coterillo, Jesús Iniesta, Angel Irabien

**Affiliations:** 1 Department of Chemical and Biomolecular Engineering, Universidad de Cantabria, Av. Los Castros s/n, 39005 Santander, Spain; 2 Department of Physical Chemistry, Institute of Electrochemistry, Universidad de Alicante, Av. Raspeig s/n, 03080 Alicante, Spain

**Keywords:** alkaline medium, CO_2_ electro reduction, chitosan, copper, ethylene production

## Abstract

This work explores the potential of novel renewable materials in electrode fabrication for the electrochemical conversion of carbon dioxide (CO_2_) to ethylene in alkaline media. In this regard, the use of the renewable chitosan (CS) biopolymer as ion-exchange binder of the copper (Cu) electrocatalyst nanoparticles (NPs) is compared with commercial anion-exchange binders Sustainion and Fumion on the fabrication of gas diffusion electrodes (GDEs) for the electrochemical reduction of carbon dioxide (CO_2_R) in an alkaline medium. They were tested in membrane electrode assemblies (MEAs), where selectivity to ethylene (C_2_H_4_) increased when using the Cu:CS GDE compared to the Cu:Sustainion and Cu:Fumion GDEs, respectively, with a Faradaic efficiency (FE) of 93.7% at 10 mA cm^−2^ and a cell potential of −1.9 V, with a C_2_H_4_ production rate of 420 µmol m^−2^ s^−1^ for the Cu:CS GDE. Upon increasing current density to 90 mA cm^−2^, however, the production rate of the Cu:CS GDE rose to 509 µmol/m^2^s but the FE dropped to 69% due to increasing hydrogen evolution reaction (HER) competition. The control of mass transport limitations by tuning up the membrane overlayer properties in membrane coated electrodes (MCE) prepared by coating a CS-based membrane over the Cu:CS GDE enhanced its selectivity to C_2_H_4_ to a FE of 98% at 10 mA cm^−2^ with negligible competing HER. The concentration of carbon monoxide was below the experimental detection limit irrespective of the current density, with no CO_2_ crossover to the anodic compartment. This study suggests there may be potential in sustainable alernatives to fossil-based or perfluorinated materials in ion-exchange membrane and electrode fabrication, which constitute a step forward towards decarbonization in the circular economy perspective.

## 1. Introduction

Innovative alternatives based on CO_2_ utilization constitute a key factor to attain the decarbonization of chemical industries [1,2]. The market price of C_2_H_4_, among other hydrocarbons, mostly compensates for the investment cost of electrochemical conversion of CO_2_ by implementation at a larger scale production [3]. The possibilities to tackle the conversion budget of CO_2_ in the energy and fossil fuel dependency timeframe have been recently surveyed [4].

The development of renewable electricity-CO_2_ derived products (RE-CO_2_DP) as a viable alternative relies on three main approaches: (i) electrocatalyst design [5]; (ii) electrode configuration; and (iii) reactor cell design and performance [6,7]. The gap between cell design and electrode configurations that must be filled before industrial production of C2+ hydrocarbons is efficient enough to be implemented at an industrial scale has been recently reviewed [8]. Copper-based electrocatalysts are the only metals known to reduce CO_2_ to C2-C3 hydrocarbons with acceptable selectivity [9,10,11]. Attempts to tune up the selectivity towards one specific hydrocarbon, i.e., C_2_H_4_ or the liquid counterpart, ethanol [12], have been undertaken by changing the surface morphology [13,14,15] and shape [16,17], reducing the particle size [18,19], directing the atomic and electronic structure of Cu [20,21,22], functionalizing nanoparticles [23,24,25,26,27,28], or supporting them into different structures such as graphene or carbon nanotubes [29,30]. Another approach focuses on coating the catalytic layer with an ion-conductive polymeric layer, such as polypyrrole (PPy), to create multifaceted Cu_2_O:PPy catalysts that reduce competitive H_2_ and CO formation in the aqueous medium [31] and modifying the functional groups attached to the Cu NPs using anionic or cationic ionomers [32] and hydrophobic or hydrophilic polymer binders [33].

The preferred electrode configuration is that of the GDEs in membrane electrode assemblies (MEAs). The basic role of the membrane in these MEAs is as a solid polyelectrolyte or ion-exchange membrane (IEM) separating the cathodic and the anodic compartments, where the electrolyte flowing through both, one, or none of the compartments leads to different types of electrochemical membrane reactors [34], usually classified as: gas–liquid (G-L) and liquid–liquid (L-L). In G-L or liquid-free type cathode configuration, the pH and the transport of species is regulated by the solid polymer electrolyte IEM separator, which has to overcome the current densities limitations posed by ion, water, and CO_2_ transport in alkaline aqueous electrolytes and the solid polyelectrolyte IEM.

Electrolytic flow reactor configurations have recently been of interest to a significant number of researchers [35,36,37]. Until recently, the most referenced divided polyelectrolyte membrane electrochemical reactors (PEMERs) focused on cation-exchange membranes (CEMs), whose benchmark were those of the Nafion^®^ family (Dupont, Wilmington, DE, USA). The outcome of commercial anion-exchange membranes (AEMs) for alkaline fuel cells opened the way to newer works claiming that AEMs allowed increasing the working pH of the alkaline electrolyte, from 1 M to 5, 7, or even 10 M [38,39,40,41]. The electrolyte media studied in the CO_2_ electroreduction to hydrocarbons on Cu surfaces is usually neutral to slightly acidic when using KHCO_3_ in aqueous media, but alkaline conditions have been observed to promote C-C bindings that, thus, shifted the selectivity to C2+ hydrocarbons and alcohols instead of HCOOH or CO [42], reducing the competition of hydrogen evolution reaction (HER) with carbon dioxide electroreduction (CO_2_R), and increasing the energy efficiency and selectivity [40,43]. Despite the risk of carbonate formation in the presence of CO_2_ flows [44,45], alkaline commercial membranes have been increasingly reported in CO_2_R, for instance FAA-3-based (Fumatech GmbH, Sankt Ingbert, Germany) [46], A201 (Tokuyama, Chiyoda City, Tokyo) [47], Selemion AMV [29,48,49], Sustainion (Dioxide Materials, Boca Raton, FL, USA) [48,49] or Aemion membranes [50] and new developments towards a higher alkaline stability are being undertaken [51].

The trade-off between HER and CO_2_R can also be tuned up by changing the composition of the ionomer binding the catalyst particles together in the catalyst layer of the gas diffusion electrode (GDE). Nwabara et al. observed that the perfluoronic acid (PFSA) ionomer content of Nafion increased the performance of GDEs as much as the metal catalyst loading, but carbonate formation was reduced by blending the Nafion ionomer with polytetrafluoroethylene (PTFE) [39]. The substitution of these cation-exchange binders by an anion-exchange Sustainion ionomer binder led to further carbonate reduction and a more stable electrode performance and lifetime [52]. Koshy et al. observed that varying imidazolium groups composition enabled the ionomer binder to control the pH of the polymer electrolyte binder to direct the CO_2_R of Ag-based electrode surfaces to H_2_ or CO [53,54]. Even though the influence of ionomer composition is mostly focused on Ag-based GDEs, recent results revealed that organic additives and ionomer types can influence the electrocatalytic activity of copper [22,28,31,50] and the selectivity of Cu-GDEs [32]. The functional groups of hydrophilic or hydrophobic polymer binders direct the electrocatalytic CO_2_R at Cu nanoparticles (NPs) towards formic acid or methane [33]. The membrane coated electrocatalysts (MCECs) approach was reviewed as a means of controlling mass transport limitations in continuous electrochemical flow reactor performances, as well as improving catalyst stability [55,56], CO_2_ permeability, and the water and ion transport limitations of the GDEs can also be overcome by coating the catalyst layer of the GDE with an ionic membrane overlayer. Coating Ag-GDEs with a Sustainion anion-exchange layer decreased the degradation of PTFE GDE by 5% [52], and modification of the electrode surface through coating with conductive polymers reduced the catalyst degradation, promoting the production of the main C product, compared to HER [57,58]. The coating of Cu-GDE with a fluorinated ethylene propylene (FEP) hydrophobic polymer binder has been reported to enhance the selectivity of C2+ hydrocarbons up to 52% in a H-reactor and 77% in a flow reactor, at −0.76 V vs. RHE [59]. The type of polymer coating can either double the Faradaic efficiency (FE) of C_2_H_4_ while maintaining the current density below 67 mA cm^−2^, or triple the current density while inhibiting CO_2_R, respectively [60]. The usual ionomer or polymer binder reported so far are based in fossil-fuel chemicals and energy intensive fabrication. Thus, a new approach is the replacing of the binder and membrane materials with more sustainable alternatives from renewable or low-cost sources with adequate ion conductivity, chemical resistance, and crossover properties.

**Figure 1:**
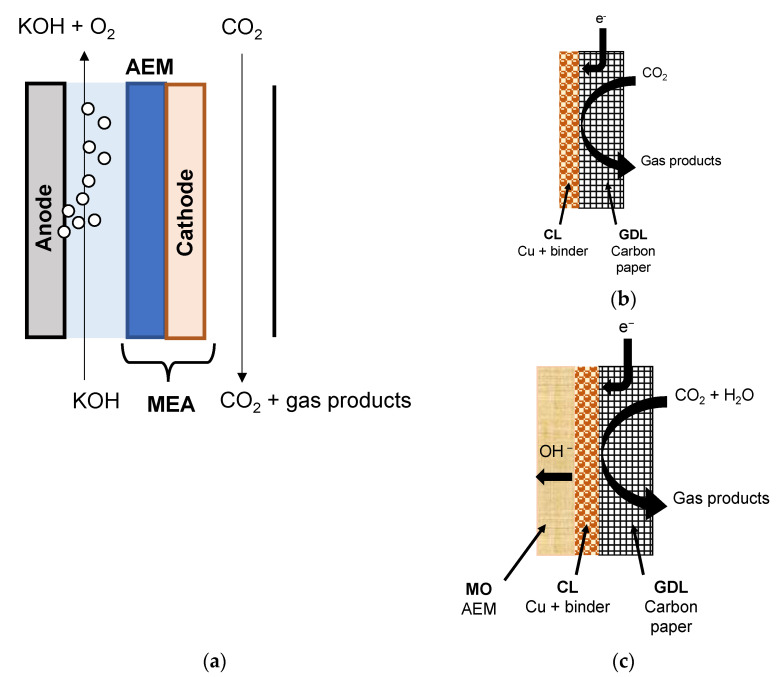
Schematic of the membrane electrode assembly configuration (MEA) for the continuous flow electrochemical cell configuration for the CO_2_R experiments (**a**), where the cathode is either of GDE architecture, with only a catalyst layer on the porous carbon support (**b**) or MCE architecture, with a membrane overlayer covering the catalyst layer (**c**), respectively. GDL: gas-diffusion layer carbon support; CL: catalyst layer; MO: membrane overlayer.

The schematic representation of the MEA configuration of the continuous flow membrane reactor, where the aqueous alkaline catholyte in the cathode compartment is replaced by a continuous humidified CO_2_ gas stream, and the solid polymer electrolyte AEM is depicted in Figure 1a, while the different architectures of the prepared GDE and membrane coated electrodes (MCE) are shown in Figure 1b,c, respectively. The difference is the effect of the membrane overlayer of the MCE on the performance of the CO_2_ reduction process to C_2_H_4_, which is also addressed in this work.

The chitosan (CS) biopolymer has been long explored as a membrane and binder material in the development of other electrochemical devices because of its renewable origin and tunable poly-anion/cation-nature [61,62,63,64]. Poly(vinyl) alcohol (PVA) is a hydrophilic, low-cost, and biodegradable polymer that has been proposed as substitute to Nafion [65] and its blending with CS is able to impart the necessary mechanical resistance without adversely affecting the ion-exchange capacity or anion conductivity [66], as well as tuning hydrophilic, ion conductivity, water transport, and ion-exchange properties [67]. In a previous work, these materials as Cu binder and membrane overlayers enabled a high FE conversion from CO_2_R to methanol in KOH 1–2 M electrolyte media [68].

In this work, the continuous CO_2_ electroreduction performance of Cu-based GDE prepared using a CS 1 wt.% solution as a binder has been compared with that of GDE prepared with commercial anion-exchange Fumion and Sustainion ionomers, for reference with the state-of-the-art. MCE and CS:PVA-based membranes whose physicochemical and electrocatalytic activity was evaluated in previous works [67,68] were introduced to study the role of the membrane overlayer. The FE and production rate of C_2_H_4_, as well as the energy efficiency, are assessed as a function of current density by using a MEA-based electrochemical continuous flow reactor.

## 2. Materials and Methods

### 2.1. Electrode Preparation

Cu-based GDEs were prepared by air-brushing using three different solutions loaded with 10 mg Cu NPs (60–80 nm, Aldrich, Madrid, Spain) in a 30:70 *w*/*w*(%) ratio in the catalyst layer, over a carbon paper sheet (Toray Carbon Paper, PTFE treated, TGP-H-60) with a thickness of 200 µm. The procedure followed is detailed in the Supplementary Materials. The GDEs were denoted as Cu:Fumion, Cu:Sustainion, and Cu:CS, as a function of the type of ionomer used, respectively. The effective geometric surface area of the GDEs was 10 cm^2^, with a catalyst loading of 1 mg cm^−2^.

The membrane coated electrodes (MCEs) were prepared by solution-casting an additional CS:PVA mixed matrix membrane layer over the Cu:CS GDE. The procedure was detailed in a previous work [68]; for specific details please go to the Supplementary Materials of the present manuscript. The membrane overlayer was composed of a polymeric blend of the CS and polyvinyl alcohol (PVA, powder, 99+% hydrolyzed, Aldrich, Spain) and a Cu-exchanged layered UZAR-S3 stannosilicate and Cu-exchanged zeolite Y, as the optimal materials for electrocatalytic reduction of CO_2_ in alkaline media tested in a prior work. The filler loading was 10 wt.% with respect to the membrane overlayer polymer volume, as the composition providing the best physicochemical and electrocatalytic results in an alkaline medium, as established in our prior works [67,68]. Please consult the Supplementary Materials for more details on the preparation and chemical and morphological characterization.

### 2.2. CO_2_R Experiments in Filter-Press Cell

The CO_2_R experiments were performed in a continuous filter-press electrochemical reactor cell (Micro Flow Cell, ElectroCell Europe A/S, Tarm, Denmark), whose flow diagram can be found in Figure S1 (Supplementary Materials). An MEA zero-gap configuration was used in the cathode chamber, as depicted before in Figure 1a. CO_2_ in a gas phase was fed directly to the cathodic compartment and reached the catalyst via the gas diffusion layer (GDL) of the electrode. A vapor delivery module (VDM) (SW-200, Bronkhorst, The Netherlands) was used to control the gas flowrate, which was established at 100 mL min^−1^, and the humidity at 1 g H_2_O h^−1^; the ratio of both reactants in the reactor was 4.8 mol CO_2_ mol^−1^ H_2_O.

The anode chamber was composed of a platinum plate as the anode, with a 1 M KOH aqueous solution as the electrolyte (KOH pellets, Panreac, Spain) flowing at a flowrate of 5.7 mL/min. An AgCl/Ag electrode was placed in the anode compartment as the reference electrode to record the anode potential. Both the anolyte and the cathode were separated by an alkaline anion exchange membrane (AAEM) previously activated in 1 M KOH. Two commercial membranes were tested: Sustainion X-37 50 grade and FAA-3, with their respective GDEs, and two CS:PVA-based membranes: a pure polymeric blend and a mixed matrix membrane (MMM) filled with Cu exchanged stannosilicate UZAR-S3 and zeolite Y, in 5 wt.% each to amount for the total 10 wt.% filler loading relative to the polymer content that led to the best physicochemical properties, as studied elsewhere [67].

The current intensity was supplied to the system using a potentiostat (MSTAT4, Arbin Instruments, College Station, TX, USA). Experiments were conducted at a fixed current intensity, settled at 100, 500, and 900 mA, respectively. The gas phase outlet at the cathode and anode compartment were carried to a micro-gas chromatograph (Inficon 3000, Agilent Technologies, Madrid, Spain). More details on the analytical procedures can be found in the Supplementary Materials.

The GDEs and MCEs were prepared from compatible ionomer, polymer, and inorganic materials, as mentioned above and detailed in the Supplementary Materials. Table 1 describes the various electrodes prepared for evaluation in this work to study the effect of the CS ion-exchange binder and the CS:PVA-based membrane overlayer upon the reaction performance. For comparison with other Cu-based electrodes reported in the literature for the electrochemical conversion of CO_2_ to C_2_H_4_ in different alkaline anolytes, please refer to Table S2 in the Supplementary Materials. The ion-exchange binder concentration and the Cu NPs loading selected in our work are in the same order of magnitude as those reported in the literature under similar conditions. The pH of the aqueous anolyte (KOH 1 M) was measured through the whole experiment and kept constant at a value close to 14.

The concentration of each product at all the current densities was averaged over three measurements. The average concentration thus obtained was applied for the calculation of the Faraday efficiency (FE) by

(1) $$\mathrm{FE}\left[\%\right]=\frac{Z\;F\;n}{q}=\frac{z\;F\;\left(c\;Q/M_{w}\right)}{i},$$

where *Z* is the number of electrons exchanged in each product reaction, *F* is the Faraday constant (96,485 C mol^−1^), *c* is the average concentration of product generated (mg L^−1^), *Q* is the volumetric flow at the outlet of the reactor (L s^−1^), *M_w_* is the molecular weight (g mol^−1^), and *i* is the total current applied to the system (*A*).

The production rates were calculated as [69,70]

(2) $$r\left[\mu \mathrm{mol}/m^{2}s\right]=\frac{c\;Q\;/M_{w}}{A},$$

where *A* is the cathode geometric area (10 cm^2^).

The energy efficiency for the generation of each product *i* (EE*_i_*) was defined as the ratio between the chemical energy stored in the product *i* and the applied electrical potential, as [71]

(3) $${\mathrm{EE}}_{i}\;\left(\%\right)=\frac{E_{0\;\mathrm{cell}}\cdot \mathrm{FE}}{E_{\mathrm{cell}}},$$

where E_0,cell_ is the standard potential of the generation of product *i* (V vs. RHE), accounting for the standard potentials at the cathode (CO_2_R) and anode (OER), FE is the Faraday efficiency (%) and E_cell_, the experimental cell potential (V).

## 3. Results and Discussion

**Figure 2:**
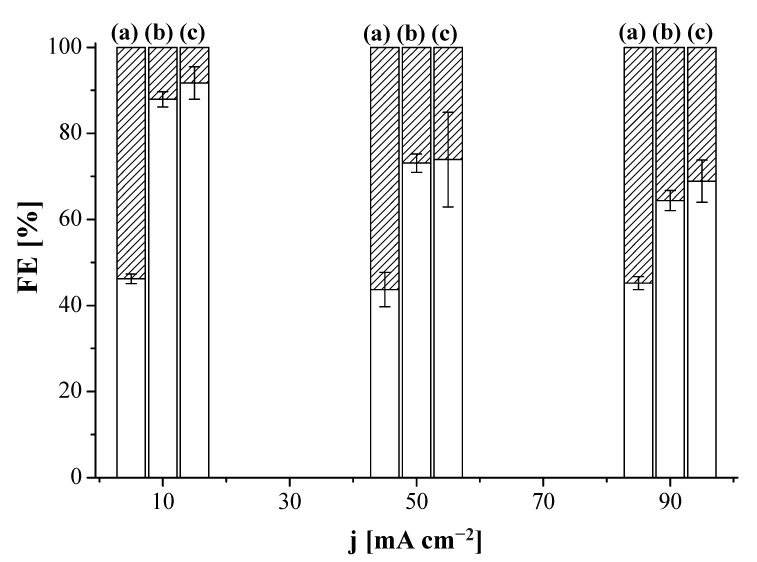
Faradaic efficiency (FE) of ethylene (blank) and hydrogen (striped) as a function of applied current density for the Fumatech MEA (FAA-3 AEM and Cu:Fumion GDE) (a), Sustainion MEA (Sustainion membrane and Cu:Sustainion GDE) (b) and the MEA composed by the Sustainion membrane and the Cu:CS GDE (c).

We first explored the influence of the AEM on the performance of the CO_2_R by using the MEA configuration in Figure 1a, to address the FE values of different products coming from the electrolysis. The performance results of CO_2_R at Cu:Sustainion GDE and Cu:Fumion GDE revealed the only products formed were C_2_H_4_ and H_2_, irrespective of the current density. Figure 2 shows the FE of C_2_H_4_ and H_2_, where the MEA configuration using the Sustainion AEM as a compartment separator outperformed the results obtained with the FAA-3 AEM. The FE for C_2_H_4_ was 87.9 ± 1.8% at 10 mA cm^−2^ and 64.4 ± 2.2% at 90 mA cm^−2^, for the CO_2_R performed at the Sustainion MEA (Sustainion AEM and Cu:Sustainion GDE), whereas for the Fumatech MEA (FAA-3 AEM and Cu:Fumion GDE), the FE was almost independent of the applied current density, being about 45% for C_2_H_4_ and 55% for H_2_. The superior OH^−^ conductivity of the Sustainion membrane (65 mS cm^−1^) [72] compared to the FAA-3 AEM (2.92 mS cm^−1^) [66] probably reduced the ohmic losses through the cell and generated a lower total cell potential. Figure S3 in the Supplementary Materials represents the overall cell potential generated at the applied current densities using the MEAs with the commercial FAA-3 and Fumion binder and the Sustainion membrane and binder, respectively. Due to the better performance of the use of Sustainion AEM, this membrane was selected in a first installment as compartment separator to build the MEA with the new Cu:CS GDE. As presented in Figure 2, the Cu:CS GDE achieved the highest selectivity to C_2_H_4_ in terms of FE. At 10 mA cm^−2^, a value of FE (C_2_H_4_) of 93.7% was attained, which monotonically decreased FE to 68.9% at 90 mA cm^−2^ due to the concomitant completion of the HER.

**Figure 3:**
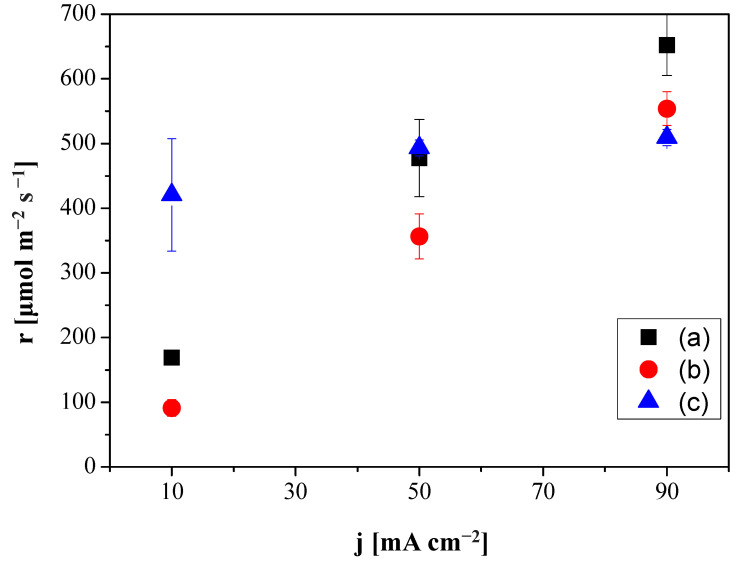
Production molar rates of ethylene generation as a function of applied current density for the Fumatech MEA (FAA-3 AEM and Cu:Fumion GDE) (a), the Sustainion MEA (Sustainion AEM and Cu:Sustainion GDE) (b) and the MEA composed by the Sustainion AEM and the Cu:CS GDE (c).

Figure 3 depicts the production rate of C_2_H_4_ as a function of current density for the CO_2_R performed at the electrodes Cu:Fumion GDE, Cu: Sustainion GDE and Cu:CS GDE, respectively. With the Sustainion MEA, the production of C_2_H_4_ ranged from 169 ± 10.3 to 652 ± 40 µmol m^−2^ s^−1^ with increasing current density from 10 to 90 mA cm^−2^, while the FAA-3 MEA generated 91.3 ± 1.3 µmol C_2_H_4_ m^−2^ s^−1^ at 10 mA cm^−2^ and 553 ± 26 mol m^−2^ s^−1^ at 90 mA cm^−2^. The reduction of HER vs. CO_2_R was less relevant with the Sustainion MEA. Nevertheless, it is worthwhile to note that the production rate of C_2_H_4_ at the Cu:CS GDE with a molar rate of 420 µmol/m^2^ s improved that of the commercial GDEs at the lowest current density (10 mA cm^−2^). Furthermore, with increasing absolute values of the current density, the production rate increase was still lower than that observed with the commercial anion-exchange MEAs but the FE(C_2_H_4_) of the latter decreased with current density and the C_2_H_4_ production rate increased monotonically, while the substitution of the alkaline commercial ionomers by CS solution led to a practically constant behavior of FE with increasing current density, as observed in Figure 2. These results agree with the recent observations made when coating the Cu NPs by hydrophilic or hydrophobic membranes [33] and the control of HER and CO_2_R by modifying the type of ionomer and alkaline conditions in Ag-GDEs [52,54].

According to the analysis of the gas phase at the cathodic compartment the main reactions expected at the cathode in this work are:

(4) $$2{\mathrm{CO}}_{2}\;\left(g\right)+8H_{2}O\left(l\right)+12e^{-}\to C_{2}H_{4}\;\left(g\right)+{12\mathrm{OH}}^{-},\;E_{0}=0.08V\;\mathrm{vs}.\;\mathrm{RHE}$$

(5) $$2H_{2}O\left(l\right)+2e^{-}\to H_{2}\;\left(g\right)+{2\mathrm{OH}}^{-},\;E_{0}=0.00V\;\mathrm{vs}.\;\mathrm{RHE}$$

In addition, the gas phase coming out of the anodic compartment was analyzed and only O_2_ was identified, which agrees well with the high pH along the whole experimental run indicating no significant carbonation of the electrolyte or appreciable carbonate crossover through the AEM barrier or CO_2_ crossover to the anode compartment, as observed by O’Brien et al. [73]. Thus, the only reaction occurring in the anode in this work is

(6) $$4{\mathrm{OH}}^{-}\to O_{2}\;\left(g\right)+2H_{2}O\left(l\right)+4e^{-},\;E_{0}=1.23V\;\mathrm{vs}.\;\mathrm{RHE}$$

Table 2 compiles the cell potential and energy efficiencies as a function of applied current density for the Cu-based gas diffusion electrodes. For comparison with other Cu-based GDE in MEA tests reported in the literature, please refer to Table S3 in the Supplementary Materials.

The energy efficiency (EE) was calculated by Equation (3) using the theorical cell standard potential of −1.15 V vs. RHE, accounting for the standard potential of the cathodic and anodic reactions, 0.08–1.23 V vs. RHE, according to reactions (4)–(6).

The largest cathode energy efficiencies reported so far for Cu-based electrodes in the electroreduction of CO_2_ to C_2_H_4_ are those presented by García-de-Alquer et al. [39], which are surpassed in our work only at 10 mA/cm^2^, for the MEA system combining the Cu:CS GDE and the Sustainion membrane. This is probably due to the negligible CO bulk formation observed in this work, which increases the selectivity towards C_2_H_4_ in the gas-phase. In fact, in our work, the analysis of the gas stream revealed CO_2_, C_2_H_4_, and H_2_ as the main products, while traces of CH_4_ and CO were detected in several experiments, but only at negligible concentrations (<<1 ppmv). CO and C_2_H_4_ were measured in different columns so overlapping of CO and C_2_H_4_ in the chromatographic analyses can be discarded. Although the conversion of CO_2_ (see Table S4 in the Supplementary Materials) was below 5%, in agreement with the literature at similar CO_2_ feed flow rates [40], the absence of CO observed in this work is relevant to minimizing further purification stages of the C_2_H_4_ stream. The CO absence is possibly due to the interplay of intermediates tuned-up by the mixed effects of the hydrophilicity [33] of the CS biopolymer binder and the influence on the metallic adsorptive properties [74] of the catalyst NPs, as well as the CO_2_ permeability towards those active sites and the resistance in alkaline media where intermediates are directed to C-H bonding [75]. The mechanism reported in hydrophilic polymer and ionomer binders leads, usually, to the CO reduction to HCOOH [33,76] and the reduction in hydrophilic character attempts to increase the selectivity of ethanol/ethylene in alkaline media [42,77,78]. For instance, coating a thin dense hydrophilic polyethylene glycol (PEG) layer decreased the formation of by-products, increasing the FE of the main product, HCOOH, up to 98%, although the production rate was as low as 0.27 µmol cm^2^ s^−1^ [79]. Although the CO_2_ flowrates were higher for the latter work, they were in the same order of magnitude as those obtained in the present work, i.e., 200–400 mL/min, and the current densities were also lower at the coated than the uncoated electrode. Thus, the introduction of tunable CS solution as binder in the preparation of Cu-based GDEs opens opportunities for improving the sustainability of the process by the substitution of toxic, fossil-based compounds by an economic and renewable alternative for the C_2_H_4_ production and selectivity of commercial anion-exchange ionomers in alkaline media.

The first report on the experimental comparison of a conventional L-L cell and a G-L half-cell achieved a total cell potential of 5 V in 0.5 M KHCO_3_ (pH 6.8), while lower than 4.2 V in 1 M KOH alkaline electrolyte. The FE towards C_2_H_4_ increased slightly (from 40–42% to 43–47% when removing the liquid KOH electrolyte from the cathode, without increasing the cell potential, at 150 mA cm^−2^ [40]. In the gas phase, though, those authors observed that CO was still produced alongside C_2_H_4_. In fact, a C_2_H_4_ selectivity peak was observed after which HER reduction was favored once more [24].

**Figure 4:**
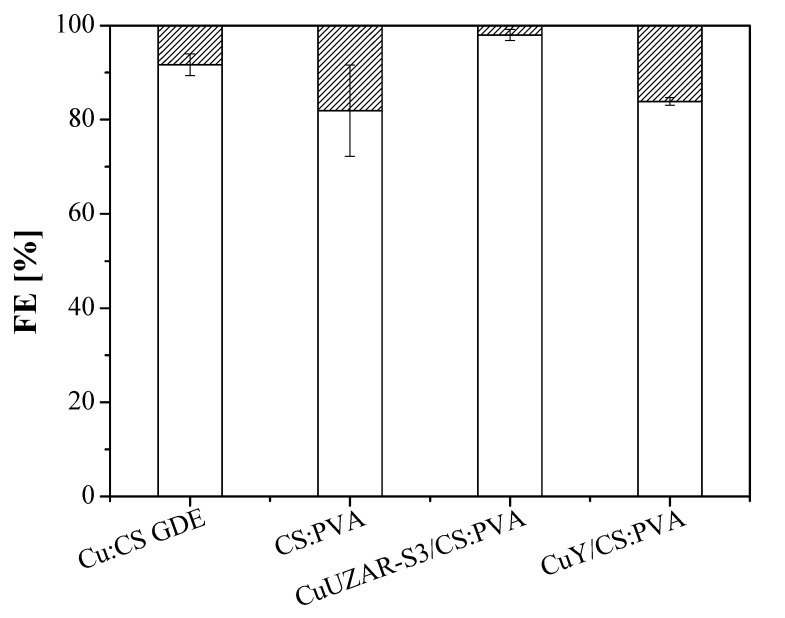
FE of ethylene (blank) and hydrogen (striped) obtained at 10 mA cm^−2^ with the three MCEs proposed in this study. The results obtained with the uncoated Cu:CS GDE are also shown for comparison. The Sustainion AEM was the compartment separator.

In our case, no CO was detected in the product stream and the use of Cu:CS GDE did not show an increase in the production rate of C_2_H_4_, as observed with the commercial anion-exchange GDE previously. The thickness of the ionomer layer coated on Cu catalysts has been reported to overcome the limited gas diffusion productivity, enhancing the cathode energy efficiency of alkaline conversion to C_2_H_4_ to values as high as 45% [39]. A correlation between the mass transfer boundary layer of the same order of magnitude as the membrane overlayer thickness in our work has been reported to pose an effect on the local CO_2_ concentration and pH near the catalyst surface [77]. Mass transport diffusion to the catalyst layer surface of the electrode is controlled by providing an OH^-^ solid interface that may eventually enhance the stability of the electrode [68,79]. For this reason, the MCEs prepared by coating the Cu:CS GDE with a pristine polymer CS:PVA or mixed matrix membrane (MMM) of tunable hydrophilic, ion-exchange, and conductive properties [67] over porous PTFE supports (the preparation conditions are included in Table 1). The FE results of these MCE together with the Cu:CS GDE commented above, using the Sustainion AEM as separator, are shown in Figure 4 towards C_2_H_4_. Table 3 compiles the cell potentials and energy efficiencies for the CO_2_R at the Cu-based MCE.

FE (C_2_H_4_) values were over 80% with an exceptional 97.98% for the electrode CuUZAR-S3CS:PVA/Cu/C MCE, which is superior to those shown by the Cu-based GDE electrodes at the same current density of 10 mA cm^−2^, and production rates for C_2_H_4_ were 270.9 ± 24.1, 284.4 ± 71. and 237.9 ± 8.5 for the electrodes CS:PVA/Cu/C MCE, CuUZAR-S3CS:PVA/Cu/C MCE, and CuYCS:PVA/Cu/C MCE, respectively. Interestingly, the production rates of C_2_H_4_ surpassed also those obtained at the Cu:Fumion GDE and Cu:Sustainion GDEs, respectively, but below the one obtained for the Cu:CS GDE with the Sustainion AEM separator.

Observing the summary of the Cu-based MCE results in Table 3, only at the lowest current density tested, 10 mA cm^−2^, the cathode potentials are comparable to those obtained with the uncoated GDEs in Table 2. This is attributed to the additional resistance provided by the membrane overlayer, whose thickness after removal from the reactor was measured at an average value of 50 µm for all three MCEs studied. This value of thickness is in the order of magnitude for similar polymer layers reported in literature (Table S2 in the Supplementary Materials), such as the 20 µm-thick Sustainion ionomer layer reported by Nwabara et al. [52] over an Ag-GDE for conversion of CO_2_ to CO, but higher than the 5.7 µm thick PFSA-based ionomer binder layer reported by Garcia-de-Alquer et al. [39], which attained one of the highest current densities of C_2_H_4_ in alkaline media reported so far. Dutta et al. [57] reported that the pore size of copper oxide electrodeposited thick films had more influence than the thickness of the film, observing that the FE decreased greatly at pore sizes below 50 µm. On the other hand, PEG electrodeposited layers of a few nm thickness, Jeong et al. [79] reached current densities lower than the uncoated electrodes, but still lower than those obtained in the present work. Most recently, Kim et al. observed for 40 nm thick Nafion and Sustainion ionomer layer on Cu-GDE that neither CO_2_ nor ion transport limitations phenomena occur at 10 mA cm^−2^ [32]. The embedding of the Cu catalyst in a conductive ionomer [39] or polymer [31] has been observed to increase the CO_2_ electroreduction performance of Cu catalyst in aqueous media, as well as improve the adhesion with the electrode substrate for the preparation of electrodes and the stability of the MEA. Consequently, in our work, ohmic losses (Figure S4 in the Supplementary Materials) for MCE are expected to occur, hindering the comprehension of the role of the membrane overlayer [56]. Thus, although the application of MCEs is promising in terms of transport facilities increasing the selectivity towards C_2_H_4_, further research is needed to improve their fabrication, especially reducing the thickness of the overlayer and limiting the ohmic losses [78], in order to apply advanced techniques as density functional theory to discern all the roles of the membrane within the reactor [33].

**Figure 5:**
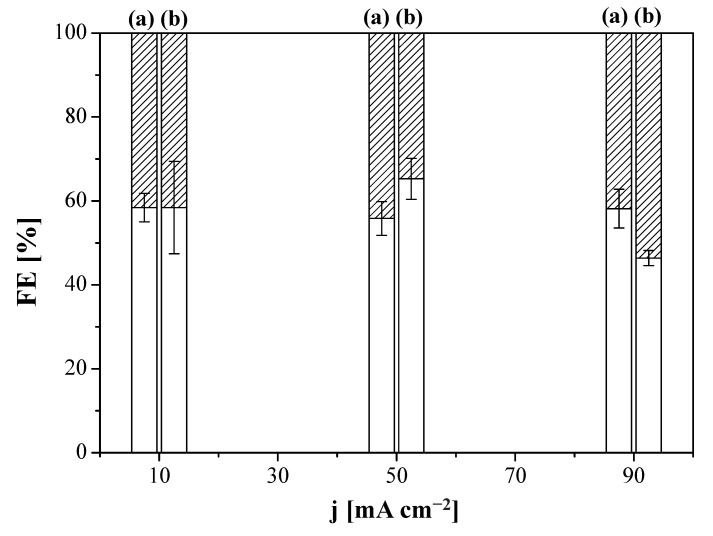
Faradaic efficiency (FE) of H_2_ (striped) and C_2_H_4_ (blank) obtained for the system with purely CS-based MEAs using the CS:PVA composite membrane (a) or the CuUZAR-S3@CuY/CS:PVA composite membrane (b) as compartment separator, as a function of applied current density. The electrode used in both cases was the Cu:CS GDE.

**Figure 6:**
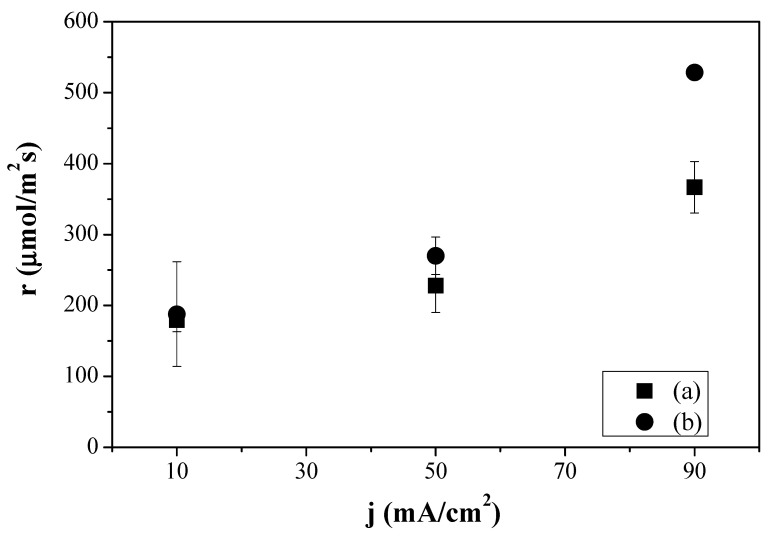
Production molar rates of ethylene obtained for the CS-based MEAs prepared in the laboratory, combining the Cu:CS GDE and the CS:PVA (a) and the CuY@CuUZAR-S3/CS:PVA MMM composite membrane (b) as a function of the applied current density.

In addition to the membrane overlayer thickness, a certain degree of material incompatibility between the CS:PVA-based membrane overlayer in the MEA composed of the prepared MCE together with the commercial Sustainion AEM as compartment separator may be the cause of additional mass transport limitations, thereby hindering the performance at high current densities. In order to verify this, we carried out several experiments replacing the Sustainion AEM in the MEA by a composite CS:PVA-based membrane, prepared by dip-coating the CS:PVA based solution onto a porous PTFE support. The cathode chosen was the Cu:CS GDE. Two composite AEMs were prepared by coating: (i) a pristine CS:PVA equimolar blend and (ii) a 5 wt.% CuUZAR-S3 and 5 wt.% CuY MMM (inorganic filler loading calculated with respect to the total polymer amount in the casting solution); it is worthwhile to note that both CuY and CuUZAR-S3 fillers provided the best synergic ion-exchange capacity, conductivity, and water transport properties to the CS:PVA based MMMs in our previous works [67,68]. The FE(C_2_H_4_) and molar production rates of the CS-based MEAs are represented in Figure 5 and Figure 6, respectively, as a function of applied current density. FE (C_2_H_4_) values were close for both MEA configurations with FE *circa* 60% and it is difficult to unveil a clear effect of current density on the FE (C_2_H_4_).

Figure 6 depicts how the C_2_H_4_ production rate highly increased with increasing current density similarly to what happened with the commercial Fumatech MEA (FAA-3 AEM and Cu:Fumion GDE) in Figure 3. Moreover, the C_2_H_4_ production rate obtained using the MEA formed by the CS:PVA composite membrane and Cu:CS GDE reached a value of 528 µmol m^−2^ s^−1^ at 90 mA cm^−2^, above even the one obtained with the MEA composed by the same Cu:CS GDE and the commercial Sustainion AEM. This indeed reveals that the compatibility of the GDE components and membrane materials, mentioned earlier, is having an effect on the overall CO_2_R efficiency.

The above results agree with some of the latest literature works reporting how substituting the conventional Nafion binder to Nafion-PTFE and Sustainion ionomer binders hindered carbonate formation in Ag- or Cu-GDE electroreduction and how the steric effects of the ionomer character alter the interaction between the ionomer ion-exchange properties and the other components of the MEA and thus facilitated diffusion to the catalyst sites [54,59]. In this work, the HER gained relevance when the Sustainion membrane (Figure 5) was replaced by a CS:PVA composite membrane (Figure 6), which was translated to lower FE(C_2_H_4_) values at all the applied current densities. This is attributed to the lower ion-exchange capacity and ionic conductivity of the CS:PVA membranes, and higher CO_2_ permeability than the Sustainion AEM [32,67]. This leads to higher cell potential along the experiment’s duration (5.01 V at 90 mA cm^−2^ for the CS:PVA based membranes, which was not attained with the Sustainion membrane, as plotted in Figure S3 of the Supplementary Materials), in agreement with Gabardo et al. [40], although the latter authors did not observe the same decrease in FE as they were working in neutral media.

A reason for the lower FE(C_2_H_4_) values may be attributed to the fact the usual two-step conversion mechanism of CO_2_ to C_2_H_4_ involving a first step conversion to CO, is not seen in this work, since no CO is observed in the gas stream, regardless the membrane or the MEA system in the reactor. The high CO_2_ permeability [80] and hydrophilicity [81] of the CS layer used as catalyst binder and polymer matrix in the MMM overlayer may account for this. Wang et al. [82] also observed that alkaline conditions increased the energy efficiency of CO and CO_2_R to C-C coupled products in Cu-based electrodes when the cathode reactions are coupled to the oxygen evolution reaction in the anode. The influence of ion transport limitations and water management constitute a key area of research in AEM since water arrives both from the aqueous anolyte, as well as the humidified CO_2_ at the cathode [83]. Because of the CO_2_ permeability and water content of the CS-based layers in GDE and MCE, we can expect that the CO_2_ and derived anions are accumulated in the anion-exchange and water swollen membrane overlayer and diffused to the active catalyst sites as reaction proceeds. The energy efficiency of the conversion of CO_2_ to C_2_H_4_ has been observed to increase with increasing pH of the reaction medium [41]. These authors also reported the lowest values of cell potential in alkaline media, as far as we know in these conditions, i.e., 2.02 V, obtained by depositing a thin polyamine layer on a Cu plate electrode, but they observed an increased CO formation diminishing product selectivity. This is supported by the calculation of the CO_2_ conversion in the same order of magnitude as other works in literature (see Table S4 in the Supplementary Materials). The only exception of a CO_2_ conversion higher than 5% reported obtained however at lower CO_2_ flow rates than this work [73]. The unreacted CO_2_ from the cell is the dominant fraction in the rest of the cases, with a small amount of carbonate crystallization in the end (see Figure S5 in the Supplementary Materials for the post-mortem SEM images of the electrodes studied in this work), which was apparently reduced by the coating of a membrane overlayer.

Although the combination of CS:PVA-coated MCEs and compatible AEMs tested in this work may show potential in the CO_2_R to C_2_H_4_, they may not become viable until other factors (membrane thickness, components interaction, compatibility, mass transport, and cathode energy efficiency) are further correlated so the internal resistance of the MCEs is reduced to the level of that of GDEs (see the electrochemical impedance spectroscopy, EIS, section and Figures S6–S8 in the Supplementary Materials).

## 4. Conclusions

Renewable materials for commercial anion-exchange binders based on oil derivatives have been explored in the preparation of electrodes for the electrochemical production of ethylene from CO_2_ at room temperature using chitosan (CS) bound copper electrodes.

The CO_2_R performance of the Cu:CS Gas Diffusion Electrode (GDE) was tested in a continuous flow cell reactor. Cu:CS GDE improved Faradaic Efficiency (FE) to ethylene compared with those obtained by the Cu:Sustainion and Cu:Fumion prepared GDEs in all the applied current ranges. In this regard, at low current density, the CO_2_R resulted in an ethylene production rate of 420 µmol/m^2^s, a FE of 93.7% with a cell potential of 1.9 V; at 90 mA cm^−2^, the production rate rose to 509 µmol m^−2^ s^−1^ and the cell potential reached 2.4 V, but the FE to ethylene dropped to 69%, due to the increase in HER.

Moreover, when the Cu:CS GDE was coated by a CuUZAR-S3/CS:PVA MMM overlayer, the selectivity of the CO_2_R to ethylene was increased further over that observed for the uncoated Cu-based GDE, up to a value of 98% at 10 mA cm^−2^ and a cell potential of 2.9 V, with an ethylene production rate of 284 µmol/m^2^s. The CO_2_R revealed the formation of ethylene and hydrogen as the only products, with significantly negligible formation of CO as intermediate.

The compatibility between the membrane and the electrode components in the Membrane Electrode Assembly (MEA) seemed to have a lesser effect on the overall reactor performance than the thickness of the membrane overlayer when the GDE is replaced by a Membrane Coated Electrode (MCE). The optimization of the electrode materials, together with the replacement and lifetime of binders and membranes is a major issue to be considered in a future work. This work opens the way to explore the potential of novel sustainable materials in membranes and electrodes for the development of ethylene production from CO_2_ in alkaline media.

## Figures and Tables

**Table 1:** Cu-based GDEs and MCEs for the production of C_2_H_4_ in KOH alkaline media prepared in our laboratory as a function of binder in the catalytic layer and the membrane overlayer composition and thickness. A 200 µm thick Toray carbon paper served as the supporting of the Cu-based electrocatalysts. References in literature in this regard are collected in Table S2 in the Supplementary Materials.

Catalyst/Cathode Type	(Ionic) Binding Type	Catalyst Loading (mg/cm^2^)	Membrane Overlayer		Reference
			Material Composition	Thickness (µm)	
Cu NP:CS/C	CS (1 wt.% in acetic acid/H_2_O)	1.0	-	-	[68]
CS:PVA/Cu NP/C	CS (1 wt.% in acetic acid/H_2_O)	1.0	CS:PVA	52 ± 1.67	[68]
CuUZAR-S3/CS:PVA/Cu-NP/C	CS (1 wt.% in acetic acid/H_2_O)	1.0	CuUZAR-S3/CS:PVA	46 ± 0.51	[68]
CuY/CS:PVA/Cu-NP/C	CS (1 wt.% in acetic acid/H_2_O)	1.0	CuY/CS:PVA	47 ± 1.98	[68]
Cu NP:S/C	Sustainion XA-9 (5 wt.% in ethanol)	1.0	-	-	This work
Cu NP:F/C	Fumion FAA-3 (10 wt.% in NMP)	1.0	-	-	This work

**Table 2:** Experimental results of the CO_2_R conversion to C_2_H_4_ in MEA configuration with Cu-based gas diffusion electrodes in 1 M KOH.

MEA Components		Anolyte	j (mA/cm^2^)	E_cat_ (V vs. RHE)	EE (C_2_H_4_) (%)
Electrode	Membrane				
Cu:Fumion GDE	Fumatech FAA-3 (AEM)	1 M KOH	10	0.57	27.2 ± 0.7
			50	0.83	20.4 ± 1.9
			90	0.92	18.6 ± 0.6
Cu:Sustainion ^1^ GDE	Sustainion X37 (AEM)	1 M KOH	10	0.48	55.2 ± 1.1
			50	0.51	42.0 ± 1.2
			90	0.62	33.0 ± 1.2
Cu:CS GDE	Sustainion X37 (AEM)	1 M KOH	10	0.53	56.4 ± 2.3
			50	0.55	41.0 ± 6.1
			90	0.74	33.5 ± 2.4
Cu:CS GDE	CS:PVA (AEM)	1 M KOH	10	0.94	10.7 ± 6.1
			50	1.96	12.0 ± 1.0
			90	2.43	10.3 ± 1.0
Cu:CS GDE	CuY@CuUZAR-S3/ CS:PVA (AEM)	1 M KOH	10	1.32	23.4 ± 4.8
			50	2.05	17.9 ± 1.3
			90	2.78	8.90 ± 0.3

^1^ The term Sustainion in the first column denotes the ionomer and the second column, the solid polyelectrolyte membrane.

**Table 3:** Experimental results of the CO_2_R conversion to C_2_H_4_ in MEA configuration with Cu-based membrane coated electrodes (MCEs) in 1 M KOH.

MEA Components		Anolyte	J (mA cm^−2^)	E_cat_ (V vs. RHE)	EE (C_2_H_4_) (%)
Electrode	Membrane				
CS:PVA/Cu/C MCE	Sustainion (AEM)	1 M KOH	10	1.26	27.8 ± 3.4
CuUZAR-S3CS:PVA/Cu/C MCE	Sustainion (AEM)	1 M KOH	10	0.87	37.8 ± 0.4
CuYCS:PVA/Cu/C MCE	Sustainion (AEM)	1 M KOH	10	0.96	13.3 ± 0.3

## Data Availability

Not applicable.

## Supplementary Materials

The following supporting information can be downloaded at: https://www.mdpi.com/article/10.3390/membranes12080783/s1, including experimental details on preparation and characterization and: Figure S1: graphical abstract; Table S1: list of acronyms and symbols used throughout the text; Table S2: Cu-based GDEs and MCEs as a function of binder in the catalytic layer reported in literature prior to our work, and the membrane overlayer composition and thickness, when available. Unless otherwise stated, the references included for comparison are those related to Cu-based electrodes for C_2_H_4_ in KOH alkaline media PEM half-cells; Table S3: CO_2_R electrochemical conversion to C_2_H_4_ in MEA with Cu-based gas diffusion and membrane coated electrodes in alkaline media reported previously in literature; Figure S2: process flow diagram of the CO_2_R experimental setup; Figure S3: cell potential vs. the applied current density applied to the continuous electrochemical reactor using the Fumatech MEA (FAA-3 membrane and Cu:Fumion GDE), the Sustainion MEA (Sustainion membrane and Cu:Sustainion GDE), as schematized in Figure 1a. Results obtained with Cu:CS GDE with the Sustainion membrane as the AEM compartment separator are also included for comparison; Figure S4: cell potential vs. the applied current density applied using the MCEs and the Sustainion AEM. Error bars represent the deviation observed for the three experimental measurements along the experiments; Table S4: comparison of the CO_2_ conversions with literature values, as a function of current densities and CO_2_ flow rates. Unless otherwise stated, the membrane barrier used in our results shown in this table is the Sustainion AEM; Figure S5: SEM images of Cu:Fumion GDE (a); Cu:Sustainion GDE (b); Cu:CS GDE (c); CuUZAR-S3CS:PVA/Cu:CS MCE (d); and CuYCS:PVA/Cu:CS MCE (e) after all the experimental runs; Figure S6: Nyquist plots of commercial Cu:Fumion GDE and Cu:Sustainion GDE, measured in Ar at a working electrode potential of −300 mV in 1 M KOH. The inset shows a zoom of the Nyquist plots at higher frequencies. Measurements were performed in triplicate; Figure S7: Nyquist plots of Toray carbon paper plate and CS:PVA-based membrane coated electrodes (MCE) measured after being saturated in Ar, at a working electrode potential of −300 mV in 1 M KOH. The inset shows a zoom of the Nyquist plots at higher frequencies. Measurements were performed in triplicate; Figure S8: partial current density of C_2_H_4_ achieved with the following MEA configurations: CS:PVA membrane + Cu:CS GDE and CuY@CuUZAR-S3/CS:PVA + Cu:CS GDE. Error bars represent the standard deviation of the FE towards C_2_H_4_ of the measurements during each experimental run. References [10,14,24,32,33,39,40,41,42,57,59,60,68,73,84,85,86,87,88,89,90,91,92,93,94] are cited in the Supplementary Materials.
