# Supplementary material for: Use of Chitosan as Copper Binder in the Continuous Electrochemical Reduction of CO2 to Ethylene in Alkaline Medium
Source: Membranes (Basel). 2022 Aug 15;12(8):783. doi: 10.3390/membranes12080783 (PMC9412364; doi:10.3390/membranes12080783)
Supplement: Supplementary file 1 [file membranes-12-00783-s001.zip › membranes-1832004-supplementary.pdf]

membranes

Supplementary information

Use of Chitosan as Copper Binder in the Continuous Electrochemical Reduction of CO_2 to Ethylene in Alkaline Medium

Aitor Marcos-Madrazo [1], Clara Casado-Coterillo [1,*], Jesús Iniesta [2] and Angel Irabien [1]

1 Department of Chemical and Biomolecular Engineering, Universidad de Cantabria, Av. Los Castros s/n, 39005 Santander, Spain
2 Department of Physical Chemistry, Institute of Electrochemistry, Universidad de Alicante, Av. Raspeig s/n, 03080 Alicante, Spain
* Correspondence: casadoc@unican.es; Tel.: +34-942-20-6777

Membrane electrode assembly for the CO_2R experiments

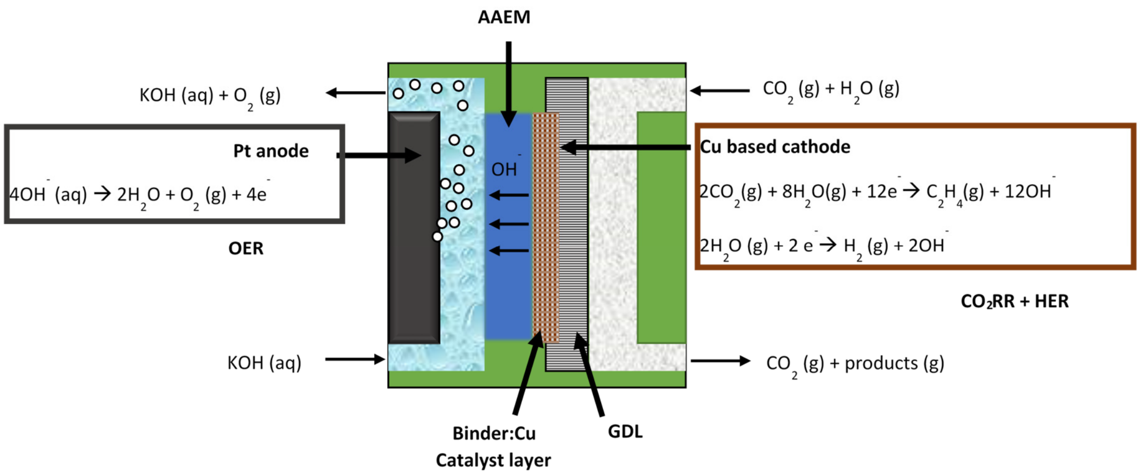

Figure S1. Graphical abstract. Membrane electrode assembly (MEA) for the CO_2R experiments. In the cathode compartment, the aqueous electrolyte was removed to build a PEMER half-cell.

Nomenclature and abbreviations

Table S1. List of acronyms and symbols used throughout the text.

Acronym	Description
A	Cathode geometric area (cm^2)

Acronym	Description
AEM	Anion exchange membrane
CEM	Cation exchange membrane
CIBH	Catalyst:ionomer bulk heterojunction (CIBH) [García-de-Alquer et al. 2020]
CL	Catalyst layer
CO_2R	CO_2 reduction
CS	Chitosan
$E_{0,cell}$	Standard cell potential (V)
E_{cell}	Cell potential (V)
EE	Energy efficiency
F	Faradaic constant, 96,485 C/mol
FE	Faradaic Efficiency
GDE	Gas diffusion electrode
GDL	Gas diffusion layer
G-L	Gas-liquid (flow cell-reactor)
HER	Hydrogen evolution reaction
i	Applied current (A)
IEM	Ion exchange membrane
IPA	isopropanol
L-L	Liquid-liquid (flow cell reactor)
MCE	Membrane-coated electrode
MEA	Membrane electrode assembly

**Acronym**	**Description**
MMM	Mixed matrix membrane
MO	Membrane overlayer (in Figure 1)
Mw	Molecular weight (g mol^{-1})
NP	Nanoparticles
OER	Oxygen evolution reaction
PEMER	Polyelectrolyte Membrane Electrochemical Reactor
PFSA	Perfluorinated sulfonic acid
PPy	Polypyrrole
PTFE	Polytetrafluoroethylene
PVA	Poly(vinyl) alcohol
Q	Volumetric flow (L/s)
r	Production rate (μmol cm^{-2} s^{-1})
RE-CO_2DP	Renewable electricity-CO_2 derived products
VDM	Vapour Delivery Module (Bronkhorst, SW-200).
z	Number of exchanged electrons

Comparison with literature

Table S2. Cu-based GDEs and MCEs as a function of binder in the catalytic layer reported in literature prior to our work, and the membrane overlayer composition and thickness, when available. Unless otherwise stated, the references included for comparison are those related to Cu-based electrodes for C_2H_4 in KOH alkaline media PEM half -cells.

**Catalyst/cathode type**	**(Ionic) binding type**	**Catalyst loading (mg/cm^2)**	**Membrane overlayer**		**Reference**
			**Material composition**	**Thickness (μm)**	
Cu wire on C paper (HNO_3)	Electrodeposition (2 C/cm^2)	0.3	Cu3,5-diamino-1,2,4-triazole (DAT)	0.70	[1,2]
Cu (100-200 nm) NP	Electrodeposition (3 C/cm^2)	0.6	-	-	[3]
Cu(100) NP	Electrodeposition	-	-	1	[4]
$Ag_{0.14}/Cu_{0.86}$ on PTFE substrate	Sputtering	0.7	-	-	[5]
Cu (150 nm) NPs/PTFE [a]	CIBH	2	-	< 6	[6]
Cu/C NP/PTFE/graphite	Nafion (5 wt% in IPA)	1.0	-	-	[7]
CuPd NP	Nafion 5 wt% (in IPA)	1.0	-	-	[8]
Cu NPs	Nafion 5 wt% (in IPA)	1.0	-	-	[9]

Catalyst/cathode type	(Ionic) binding type	Catalyst loading (mg/cm^2)	Membrane overlayer		Reference
			Material composition	Thickness (μm)	
Cu-MOF/nanographene	Nafion 117 (5 wt% in IPA)	1.1	-	-	[10]
Cu NPs	Fluorinated ethylene polymer (FEP)	0.8	-	$1.2 \cdot 10^{-3}$	[11]
Cu NPs	Nafion 5 wt% (in IPA)<br>Poly(acrylic acid) (PAA)<br>Polyvinylidene difluoride (PVDF)	0.5	-		[12]
Cu-SiOx/PTFE	Aquivion (D79-25BS)	1.25	-	-	[13]
Cu NPs	Nafion 5 wt% (in IPA)<br>Sustainion ionomer (5 wt% in ethanol)	$6 \cdot 10^{-3}$	Nafion 117<br>Sustainion	$30 \cdot 10^{-3}$<br>$40 \cdot 10^{-3}$	[14]
PFSA/Cu/PTFE	Nafion (5 wt% in IPA)	3.33	PFSA[c]	5.7	[15]
Cu-foam [b]	Polymer-modified electrodeposition	0.25	Polyacrylamide	3	[16]
Cu-foam/Silica wafer	Electrodeposition	25.3	PVD/CuO	85	[17]
Cu/PTFE	Electrochemical/sol-gel	0.7%[c]	TiO_2	0.05	[18]

Catalyst/cathode type	(Ionic) binding type	Catalyst loading (mg/cm^2)	Membrane overlayer		Reference
			Material composition	Thickness (μm)	
Cu plate	Co-electrodeposition	1.0	Polyamine	2	[19]

[a] the anolyte is KOH 7 M; [b] the anolyte is $NaHCO_3$ 0.5 M; [c] Cu-layer thickness to PTFE support thickness ratio.

Table S3. CO_2R electrochemical conversion to C_2H_4 in MEA with Cu-based gas diffusion and membrane coated electrodes in alkaline media reported previously in literature.

MEA components		Anolyte	j (mA/cm^2)	E_{cat} (V vs RHE)	FE(C_2H_4) (%)	r (C_2H_4) (μmol/m^{2}s)	EE(C_2H_4) (%)	Reference
Electrode	Membrane							
Cu wire	Fumatech FAP-375-PP (CEM)		300	0.7	60	793 [a]	51	[2]
Cu NP (100-200 nm) (GDE)	Fumatech FAA-3 (AEM)		653	0.67	38.6	1249	37.8 [a]	[3]
Cu/PTFE (GDE)	Sustainion X37 (AEM)	7 M KOH	150	3.25-4.2	46 - <40	N/A	15	[6]
Cu foam (GDE)	Nafion 117 (CEM)	0.5 M $NaHCO_3$	5-7	0.6	21	N/A	N/A	[17]

MEA components		Anolyte	j (mA/cm²)	E_{cat} (V vs RHE)	FE(C_2H_4) (%)	r (C_2H_4) (µmol/m²s)	EE(C_2H_4) (%)	Reference
**Electrode**	**Membrane**							
Cu foam (GDE)	Nafion NRE 212 (CEM)	0.5 M $NaHCO_3$	>20	0.96	13	N/A	N/A	[16]
Cu (100nm)/CNT/ PTFE/graphite (GDE)	Nafion (CEM)	7 M KOH	75	0.54	70	N/A	34	[7]
Cu_2S (GDE)	Nafion 117 (CEM)	1 M KOH	120	0.95	19±2	N/A	11.8	[20]
CuPd NP:Nafion (GDE)	Fumatech FAA-3 (AEM)	1 M KOH	360.5	0.71	48	N/A	N/A	[8]
Cu NP (20-40nm):Nafion (GDE)	Fumatech FAA-3 (AEM)	1 M KOH	413	0.58	35	615	30	[9]
Cu:PTFE (GDE)	Fumasep FAA-3-PK-130 (AEM)	1 M KOH	120.75	0.67	48.3±5.5	N/A	20	[18]
F-Cu/Nafion (GDE)	Neosepta AHA (AEM)		800<br>1200	0.54<br>0.89	60<br>65	2106<br>4013	55<br>44	[21]

MEA components		Anolyte	j (mA/cm^2)	E_{cat} (V vs RHE)	FE(C_2H_4) (%)	r (C_2H_4) (µmol/m^{2}s)	EE(C_2H_4) (%)	Reference
**Electrode**	**Membrane**							
$Ag_{0.14}$:$Cu_{0.86}$- (150nm) PTFE/PP (GDE)	Fumasep FAA-3-PK-130 (AEM)	1 M KOH	250	0.67	41	N/A	24.7	[5]
Polyacrylamide/Cu foam (MCE)	Nafion NRE 212 (CEM)	0.1M $NaHCO_3$	>20	0.96	26	N/A	N/A	[16]
PFSA/Cu/PTFE (MCE)	Nafion (CEM)	7M KOH	800	N/A	60	N/A	20-40	[15]
Polyamine/Cu plate	N/A	1M KOH<br>10M KOH	312	0.77<br>0.47	90<br>87	N/A	50	[19]
Cu:FEP (GDE) [d]	Nafion 212 (CEM)	1M KOH	600	0.76	77 [a]	N/A	N/A	[11]

[a] Values referred to C^{2+} (ethylene and ethanol) production; [b] cell potential; [c] the term Sustainion in the first column denotes the ionomer and the second column, the solid polyelectrolyte membrane; [d] FEP: fluorinated ethylene polymer binder.

Electrode preparation

Materials and methods

Commercial Cu nanoparticles (60-80 nm, Sigma Aldrich, Spain) were employed as catalyst in the fabrication of GDEs. Three different ionomer solutions were studied as binder for the preparation of the Cu-based GDEs: commercial alkaline ionomers Fumion® FAA-3 (10 wt.% in NMP, Fumatech) and Sustainion® (5 wt.% in ethanol, Dioxide Materials), and CS 1 wt.% solution in a 2 wt.% acetic acid aqueous solution. TGP-H-60 Toray Carbon Paper, PTFE-treated, with a thickness of 200 μm was used as porous support.

The catalytic ink used in the fabrication of the catalyst layer, CL, contained 10 mg of Cu NPs, in the calculated a certain amount of each binder solution (depending on its wt. concentration) to ensure a catalyst:binder ratio of 70:30 (w/w%), and a solvent, isopropanol with the commercial ionomers, and ultrapure water, when CS was used as binder. In all three cases, the mixture was sonicated in an ultrasonic bath (Selecta ultrasonic bath, 50/60 kHz) for 30 min before being airbrush coated on the carbon support. The effective geometric surface area of the GDEs was 10 cm^2, with a catalyst loading of 1 mg cm^{-2}. The GDEs were denoted as Cu:Fumion GDE, Cu:Sustainion GDE and Cu:CS GDE, respectively.

Membrane Coated Electrodes (MCEs), on the other hand, were prepared by the solution-casting of an additional CS:PVA mixed matrix membrane (MMM) over layer on top of the Cu:CS GDE, which was detailed elsewhere [22]. In short, this MMM overlayer was prepared by blending a 1 wt.%. CS solution with a 4 wt.% poly(vinyl) alcohol (PVA, powder, 99+% hydrolyzed, Sigma Aldrich, Spain) solution, the resulting in an equimolar polymeric blend. This solution was cast directly on the surface of the Cu:CS GDE with the aid of a doctor blade. The aqueous solvent was evaporated at room temperature in a fume hood to constant weight. Then the MCE was activated by immersion in a 1 M KOH solution for 1 h, then rinsed the excess of alkaline solution in abundant distilled water. Three different MCEs were prepared by modifying the overlayer composition: a CS:PVA polymer membrane overlayer, and two (MMM overlayers where the CS:PVA polymer matrix was loaded with Cu-ion-exchanged zeo-type fillers: CuUZAR-S3 and CuY.

The process flow diagram of the CO_2R experimental setup is depicted in Figure S2. The flow rate of the 1 M KOH anolyte is 5.7 mL min^{-1}. The potentiostat gives the anode potential vs Ag/AgCl and the cell potential is measured with a multimeter. The cathode potential is calculated as [23],

$$E_{cat} vs\ Ag/AgCl = E_{cell} + E_{an} vs\ Ag/AgCl - \eta_{ohm} \quad \text{(S1)}$$

where the ohmic overpotential is calculated as the sum of the ohmic overpotential of the anolyte solution and the commercial membrane barrier, as

$$\eta[V] = \frac{\delta(cm) \cdot j\left(\frac{mA}{cm^2}\right)}{\sigma\left(\frac{S}{cm}\right) \cdot 1000} \quad \text{(S2)}$$

where δ is the anolyte thickness or gap between the membrane and the anode, 0.4 cm, or the AEM barrier thickness, respectively, and σ the specific conductivity of the anolyte electrolyte (0.2009 S cm^{-1}) and AEM, respectively (65 mS cm^{-1} and 3 mS cm^{-1} for the Sustainion and FAA-3 AEM, respectively).

The cathode potential included in Table 2 from the main text is expressed vs RHE to account for the alkaline pH of the cell by

$$E_{cat} vs\ RHE = E_{cat} vs\ Ag/AgCl + 0.197 + 0.059 \cdot \mathrm{pH} \quad \text{(S3)}$$

PEMER set-up

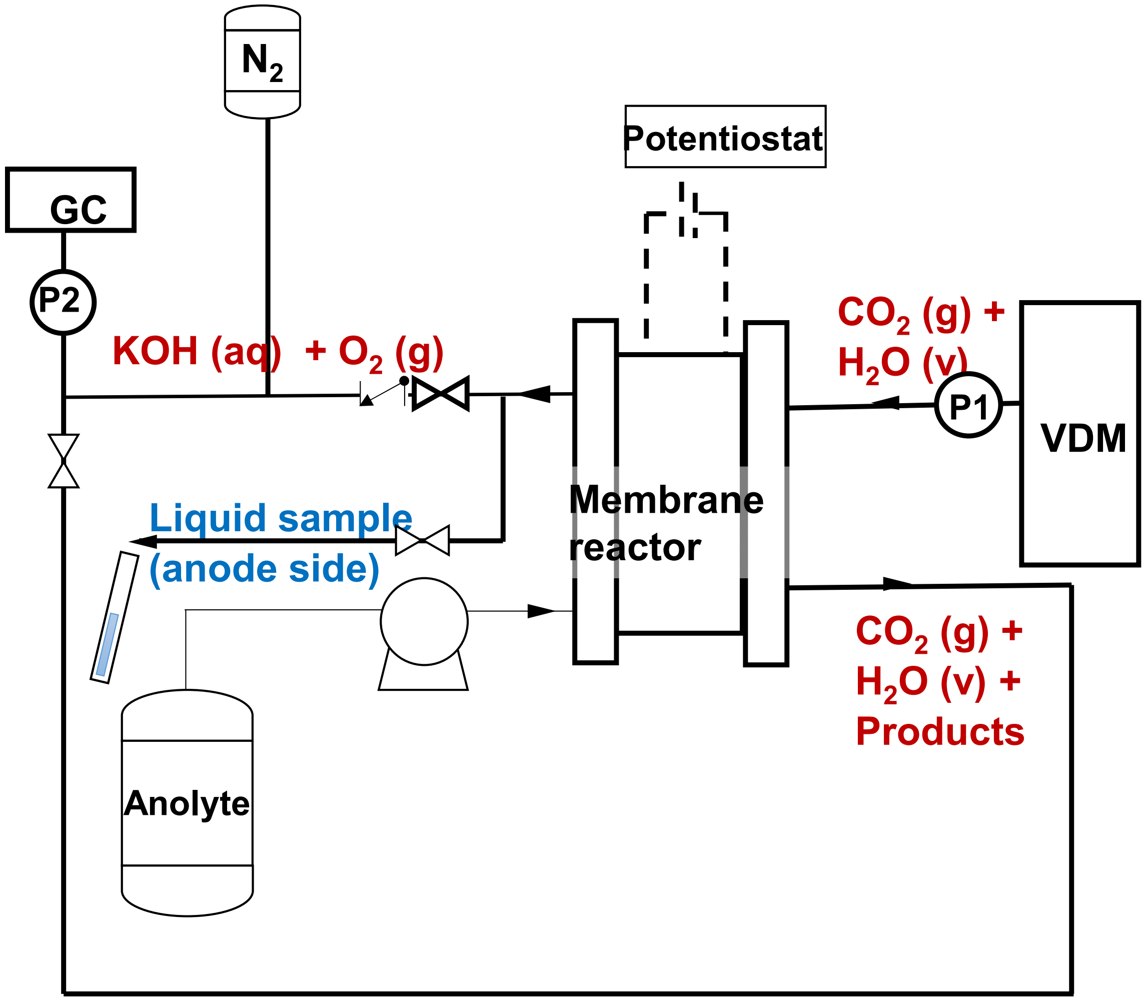

Figure S2. Process flow diagram of the CO2R experimental setup.

Plots of cell voltage versus current density

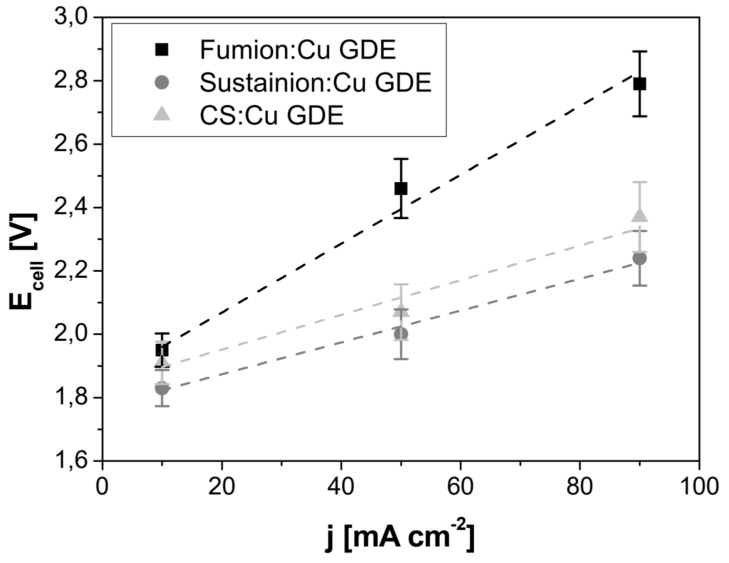

Figure S3. Cell potential vs the applied current density applied to the continuous electrochemical reactor using the Fumatech MEA (FAA-3 membrane and Cu:Fumion GDE), the Sustainion MEA (Sustainion membrane and Cu:Sustainion GDE) as schematized in Figure 1(**a**). Results obtained with Cu:CS GDE with the Sustainion membrane as AEM compartment separator is also included for comparison.

The slope of the curves corresponds to the internal resistance of the electrochemical reactor, having values of 10.86 ± 1.13 Ω cm^2, 5.02 ± 0.01 Ω cm^2 and 5.47 ± 0.01 Ω cm^2 for the Cu:Fumion, Cu:Sustainion and Cu:CS GDE. The difference between the fully commercial MEAs is due to the membrane and ionomer conductivity differences, thus it is remarkable the similarities of the internal resistance observed when substituting the Sustainion ionomer by CS 1 wt.% solution, where the cell potential at zero current density is closer to the FAA-3-Cu:Fumion MEA than the Sustainion-Cu:Sustainion MEA, despite the difference in the membrane used as barrier between the compartments.

The intercept of the linear fitting give values of 1.852 V, 1.773V and 1.742 V for the Cu:Fumion, Cu:Sustainion and Cu:CS GDE in this case, which corresponds to the values of the open circuit voltage (OCV) at j = 0.

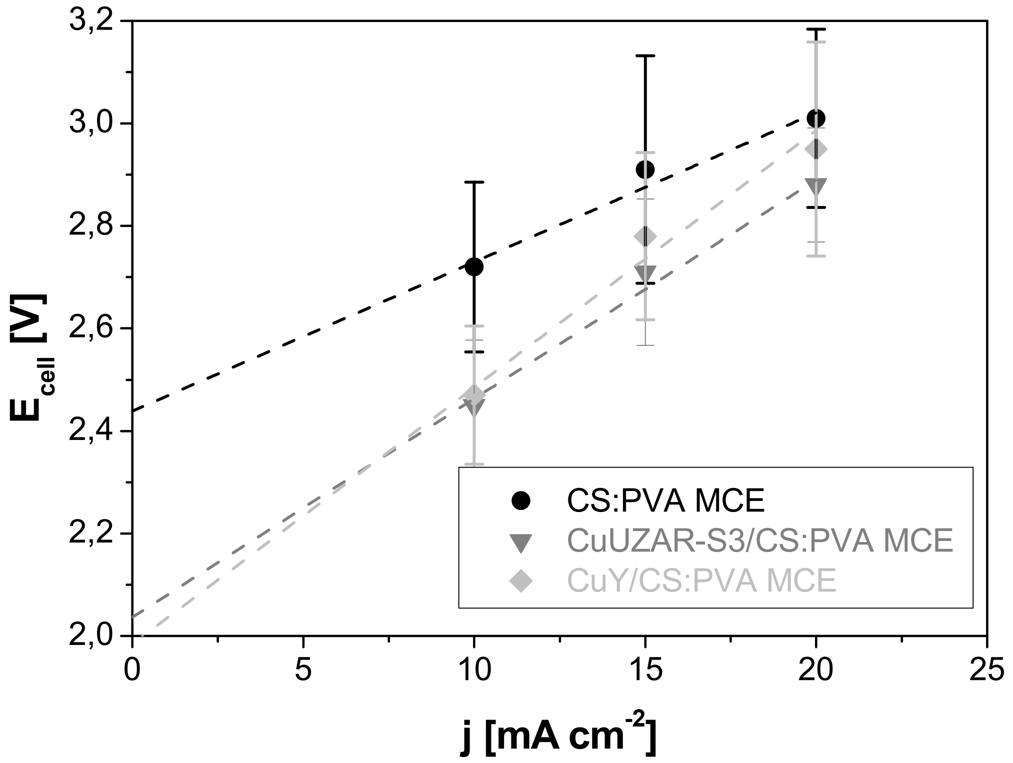

Figure S4. Cell potential vs the applied current density applied using the MCEs and the Sustainion AEM. Error bars represent the deviation observed for the three experimental measurements along the experiments.

The slope has been extended to estimate the OCV of the electrochemical reactor, which is significantly higher when the membrane overlayer is made of pristine CS:PVA polymer blend (2.44 V) than CuUZAR-S3/CS:PVA (2.04 V) and CuY/CS:PVA (1.98 V) MMM overlayer, respectively.

From the slope of the curves, the internal resistance of the CS:PVA, CuUZAR-S3/CS:PVA and CuY/CS:PVA MCEs are 29.4, 43 and 49 Ω cm^2. The internal resistance of the MCE configurations are almost 10-fold in order of magnitude of the GDE without membrane overlayer, which is consistent with the experimental results reported above and in the main text.

Analytical measurements

The experiments with every cathode configuration were run for 60 min at least twice for reproducibility reasons. The gas stream leaving the cathodic compartment was carried to a microGC (Inficon 3000, Agilent Technologies) equipped with the following columns and a thermal conductivity detector (Micro-TCD):

A) 10 m Molsieve/3 m Plot U, 1.0 μL Backflush Injector, carrier gas: Argon

B) 12 m Plot Q/1 m Plot Q, 1.0 μL Backflush, carrier gas: Helium

C) 10 m OV-1 2.0 μm thick/1.2 m Stabilwax 1.0 μL Backflush Injector, carrier gas: Helium

D) 14 m Stabilwax/1.2 m Stabilwax, 1.0 μL Backflush Injector, carrier gas: Helium

Since H_2, CO, CH_4, N_2, O_2 and some noble gases were detected in column A, this allowed the identification of the H_2 generated due to the undesired HER that takes place in the cathode. Although small peaks representing CO and CH_4 were detected in a few experiments, with concentrations below the sensitivity detection of the equipment, about << 1 ppm of these gases, so they were considered as a negligible product in this work. Additionally, the gas phase of the anode was analyzed in column A. An auxiliary nitrogen stream was employed to carry the gas phase with enough pressure to the microGC. Only O_2 (and the N_2 as carrier) was detected as gas product of the anode side.

CO_2 and larger chain hydrocarbons were separated in column B, which returned the concentration of CO_2 and C_2H_4. Columns C and D, for Volatile solvents and polar compounds, respectively, were not necessary in this work.

Calculation of CO_2 conversion

The conversion of CO_2 to ethylene has been calculated for the GDEs prepared with Fumion, Sustainion and CS binders, respectively, in the membrane electrode assemblies with the commercial FAA-3 and Sustainion AEMs, respectively, according to the equation:

$$X(CO_2)[\%] = \frac{n(CO_2)_{consumed}}{n(CO_2)_0} \times 100$$

where n(CO_2) represents the moles of CO_2 consumed and introduced to the reactor per mole of C_2H_4 generated, according to sub-indexes "consumed" and "0", respectively. The flow rate of CO_2 was 52 cm^3(STP) min^{-1} in all experiments, which leaves 0.27 mol CO_2 in 60 min.

Table S4. Comparison of the CO_2 conversions with literature values, as a function of current densities and CO_2 flow rates. Unless otherwise stated, the membrane barrier used in our results shown in this table is the Sustainion AEM.

j (mA/cm^2)	CO_2 flow rate (cm^3 (STP) min^{-1})	CO_2 conversion (%)	Reference
90	52	5.9	This work (Cu:Fumion GDE, FAA-3 membrane)
90	52	7.0	This work (Cu:Sustainion GDE)
90	52	5.4	This work (Cu:CS GDE)
90	52	3.9	This work (Cu:CS GDE, CS:PVA membrane)
90	52	5.6	This work (Cu:CS GDE, MMM)
10	52	1.9	This work (CS:PVA MCE)
10	52	3.0	This work (CuUZAR-S3/CS:PVA MCE)
10	52	7.0	This work (CuY/CS:PVA MCE)

j (mA/cm^2)	CO_2 flow rate (cm^3 (STP) min^{-1})	CO_2 conversion (%)	Reference
50	40	5.0	[6]
100	1<br>10	85<br>20	[24]
12.6	16	8.0	[25]

Scanning electron microscopy of the surface of the electrodes

SEM images of the electrodes were recorded in a microscope HITACHI S-3000N coupled with an energy dispersion X-ray microanalysis EDX system Quantax 400 from Bruker.

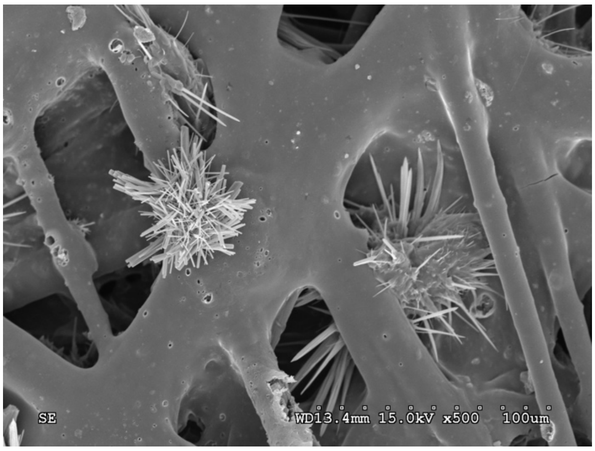

(a)

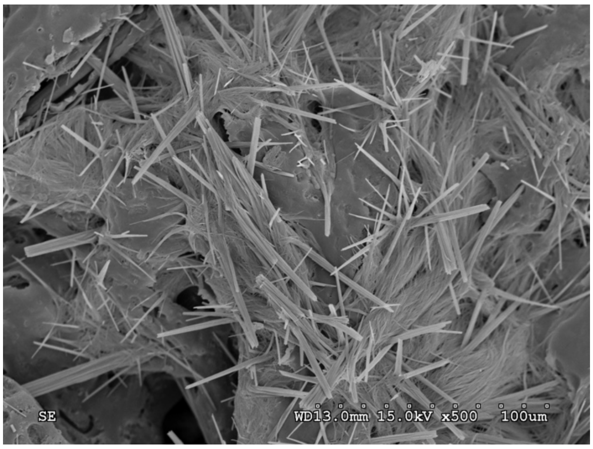
SE
WD13.0mm 15.0kV x500 100um

(b)

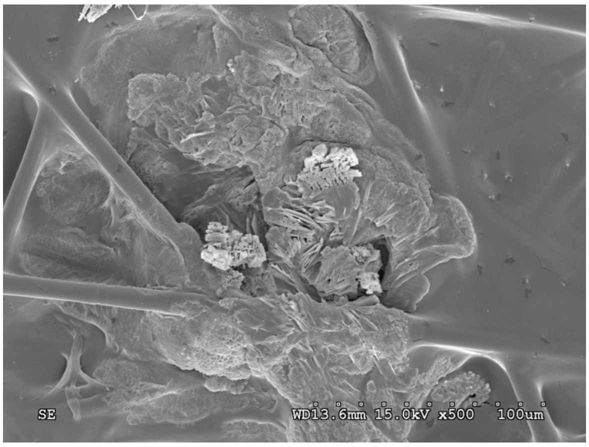
SE
WD13.6mm 15.0kV x500 100um

(c)

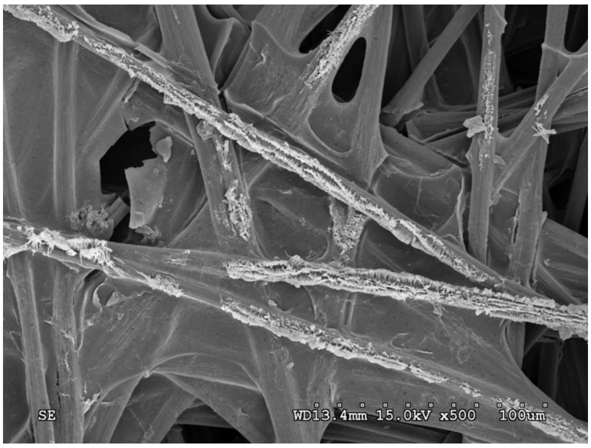
SE
WD13.4mm 15.0kV x500 100um

(d)

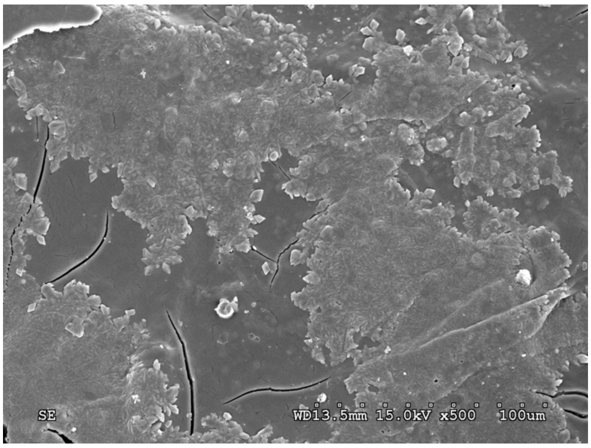

(e)

Figure S5. SEM images of Cu:Fumion GDE (**a**); Cu:Sustainion GDE (**b**); Cu:CS GDE (**c**); CuUZAR-S3CS:PVA/Cu:CS MCE (**d**) and CuYCS:PVA/Cu:CS MCE (**e**) after all the experimental runs.

Electrochemical Impedance Spectroscopy

Electrochemical impedance spectroscopy (EIS) experiments were performed using a Bio-logic system equipped with an impedance module at open circuit potential (potentiostatic method). A three electrochemical cell was used where a gold wire and AgCl/Ag (3.5 M KCl) electrodes were used as counter and reference electrode, respectively. A *ca.* 5 mm x 5 mm GDE or MCE based electrodes were used as the working electrodes. EIS experiments were carried out in 1 M KOH under saturated Ar. The working potential was set at the open circuit potential with an amplitude of 10 mV and the frequency range was varied between 1 MHz and 100 mHz.

EIS measurements were carried out in order to correlate the internal resistances of the electrochemical reactor which are associated with the electrolyte resistance, the membrane resistances and the electron transfer and mass transport resistance of the distinct cathodes, *i.e.*, GDE and MCE based electrodes.

Figures S6 and S7 depict the Nyquist plots for GDE and MCE based electrodes, respectively, under Ar saturated solution in 1 M KOH.

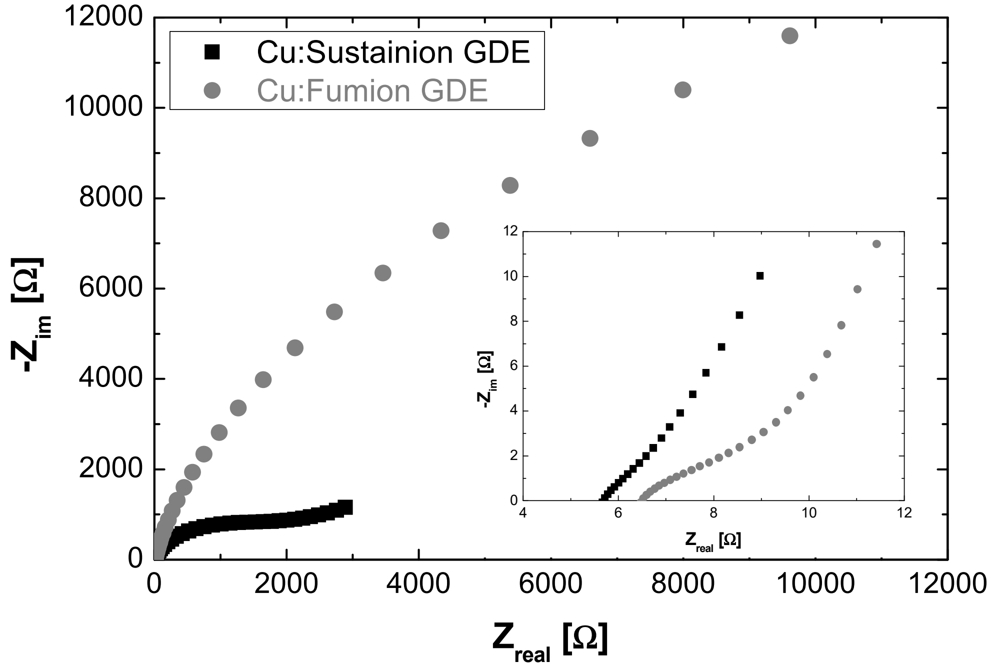

Figure S6. Nyquist plots of commercial Cu:Fumion GDE and Cu:Sustainion GDE, measured in Ar at a working electrode potential of -300 mV in 1 M KOH. The inset shows a zoom of the Nyquist plots at higher frequencies. Measurements were performed in triplicate.

The contribution of the bulk solution resistance plus the resistance associated to ionomer/layer on the underlying substrate carbon paper (R_s) can be estimated at the highest frequency, namely, the high frequency intercept of the impedance spectrum. The much smaller value for the Sustainion based MEA (5.7 Ω, 1.43 Ω cm^2) than that for the FAA-3 based MEA (6.4 Ω, 1.60 Ω cm^2) is indicative of the lower internal resistance obtained from Figure S3 for the Sustainion based MEA. The above differences in resistances is mostly due to the membrane resistance (note that during electrosynthesis operations the membrane is fully hydrated and CO_2 is fully humidified so it is assumed that internal resistance do not change within the interval range of current density). Moreover, the Nyquist plot in Figure S6 reveals that the Sustainion-based GDE offered the lowest semicircle associated to the polarization charge transfer (R_P) or activation compared to that for the FAA based GDE. The above behavior is also in agreement with the results obtained in Figure S3, where the use of the Sustanion-based GDE reduces the internal resistance of the electrochemical cell, being

understood the internal resistance the sum of two polarizations such sum of activation and diffusion polarizations.

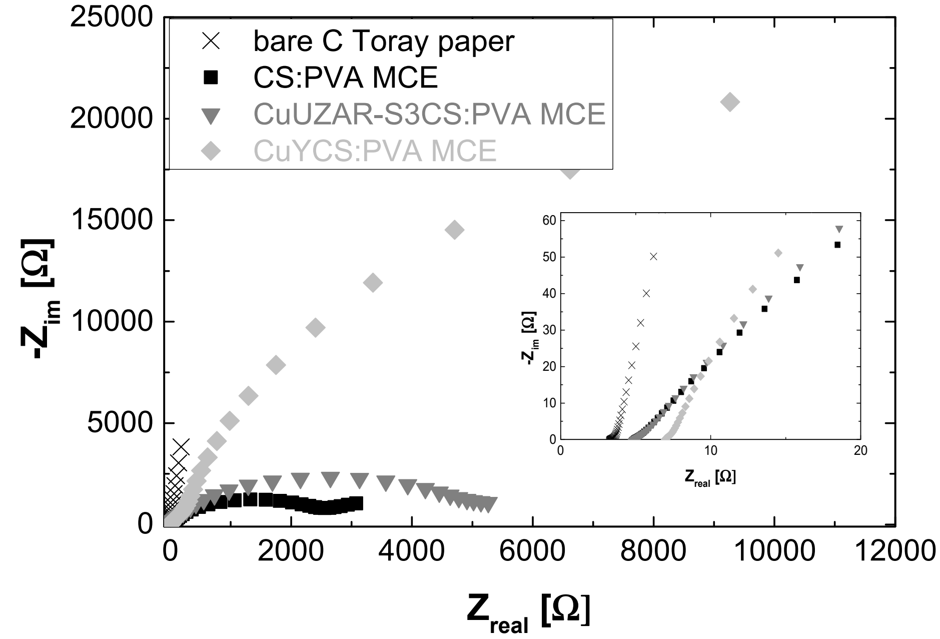

Figure S7. Nyquist plots of Toray carbon paper plate and CS:PVA-based membrane coated electrodes (MCE) measured after being saturated in Ar, at a working electrode potential of -300 mV in 1 M KOH. The inset shows a zoom of the Nyquist plots at higher frequencies. Measurements were performed in triplicate.

The Nyquist plots obtained when using the MCE based electrodes reveals that the CuY/CS:PVA MCE electrode has a R_s value of 7.0 Ω (2.80 Ω cm^2) which is the highest resistance value of the series, with a value of 4.9 Ω, on the other hand, for the CS:PVA MCE (1.96 Ω cm^2) and CuUZAR-S3CS:PVA/Cu:CS MCE (1.96 Ω cm^2), respectively. Samples based on MCE show a wide, remarkable semicircle associated to the charge transfer and mass transport resistances with an increasing trend of CS:PVA< CuUZAR<CuY loaded MMM in MCE. The above trend is also a consequence of the highest internal resistances' values obtained from the plot of cell potential versus current density plots in Figure S4.

The use of an equivalent circuit based on (R_s) in series with possibly two parallel constant phase elements (CPE) for the evaluation of quantitative data was unsuccessful due

to the complexity of reaction kinetics on the GDE and MCE electrodes interface in solution. Hence, EIS measurements will be refined in PEM -half cell electrochemical reactor configuration in a future work.

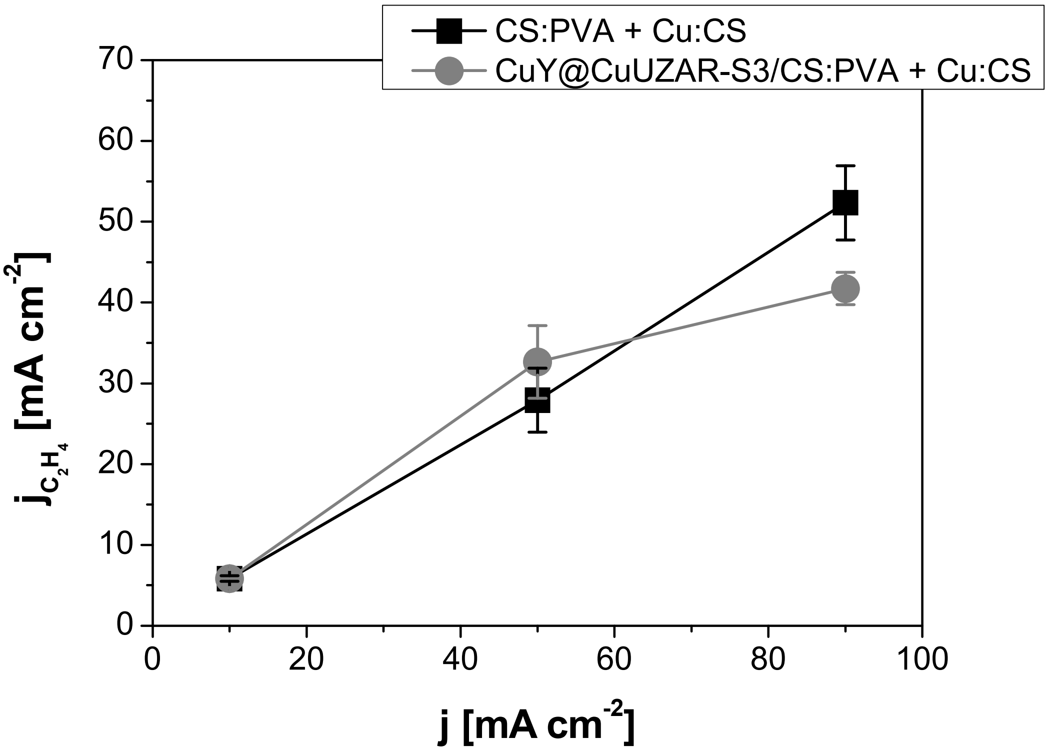

Figure S8. Partial current density of C_2H_4 achieved with the following MEA configurations: CS:PVA membrane + Cu:CS GDE and CuY@CuUZAR-S3/CS:PVA + Cu:CS GDE. Error bars represent the standard deviation of the FE towards C_2H_4 of the measurements during each experimental run.

References

1. Hoang, T.T.H.; Ma, S.; Gold, J.I.; Kenis, P.J.A.; Gewirth, A.A. Nanoporous Copper Films by Additive-Controlled Electrodeposition: CO2 Reduction Catalysis. *ACS Catal.* **2017**, *7*, 3313–3321, doi:10.1021/acscatal.6b03613.
2. Hoang, T.T.H.; Verma, S.; Ma, S.; Fister, T.T.; Timoshenko, J.; Frenkel, A.I.; Kenis, P.J.A.; Gewirth, A.A. Nanoporous Copper-Silver Alloys by Additive-Controlled Electrodeposition for the Selective Electroreduction of CO2 to Ethylene and Ethanol. *J. Am. Chem. Soc.* **2018**, *140*, 5791–5797, doi:10.1021/jacs.8b01868.
3. Lv, J.J.; Jouny, M.; Luc, W.; Zhu, W.; Zhu, J.J.; Jiao, F. A Highly Porous Copper Electrocatalyst for Carbon Dioxide Reduction. *Adv. Mater.* **2018**, *30*, 1–8, doi:10.1002/adma.201803111.
4. Wang, Y.; Wang, Z.; Dinh, C.T.; Li, J.; Ozden, A.; Golam Kibria, M.; Seifitokaldani, A.; Tan, C.S.; Gabardo, C.M.; Luo, M.; et al. Catalyst Synthesis under CO2 Electroreduction Favours Faceting and Promotes Renewable Fuels Electrosynthesis. *Nat. Catal.* **2020**, *3*, 98–106, doi:10.1038/s41929-019-0397-1.
5. Li, Y.C.; Wang, Z.; Yuan, T.; Nam, D.H.; Luo, M.; Wicks, J.; Chen, B.; Li, J.; Li, F.; De Arquer, F.P.G.; et al. Binding Site Diversity Promotes CO2 Electroreduction to Ethanol. *J. Am. Chem. Soc.* **2019**, *141*, 8584–8591, doi:10.1021/jacs.9b02945.

6. Gabardo, C.M.; O'Brien, C.P.; Edwards, J.P.; McCallum, C.; Xu, Y.; Dinh, C.T.; Li, J.; Sargent, E.H.; Sinton, D. Continuous Carbon Dioxide Electroreduction to Concentrated Multi-Carbon Products Using a Membrane Electrode Assembly. *Joule* **2019**, *3*, 2777–2791, doi:10.1016/j.joule.2019.07.021.
7. Dinh, C.T.; Burdyny, T.; Kibria, G.; Seifitokaldani, A.; Gabardo, C.M.; Pelayo García De Arquer, F.; Kiani, A.; Edwards, J.P.; De Luna, P.; Bushuyev, O.S.; et al. CO2 Electroreduction to Ethylene via Hydroxide-Mediated Copper Catalysis at an Abrupt Interface. *Science (80-.).* **2018**, *360*, 783–787, doi:10.1126/science.aas9100.
8. Ma, S.; Sadakiyo, M.; Heim, M.; Luo, R.; Haasch, R.T.; Gold, J.I.; Yamauchi, M.; Kenis, P.J.A. Electroreduction of Carbon Dioxide to Hydrocarbons Using Bimetallic Cu-Pd Catalysts with Different Mixing Patterns. *J. Am. Chem. Soc.* **2017**, *139*, 47–50, doi:10.1021/jacs.6b10740.
9. Ma, S.; Sadakiyo, M.; Luo, R.; Heima, M.; Yamauchi, M.; Kenis, P.J.A. One-Step Electrosynthesis of Ethylene and Ethanol from CO2 in an Alkaline Electrolyzer. *J. Power Sources* **2016**, *301*, 219–228, doi:10.1016/j.jpowsour.2015.09.124.
10. Zhi, W.Y.; Liu, Y.T.; Shan, S.L.; Jiang, C.J.; Wang, H.; Lu, J.X. Efficient Electroreduction of CO2 to C2-C3products on Cu/Cu2O@N-Doped Graphene. *J. CO2 Util.* **2021**, *50*, 101594, doi:10.1016/j.jcou.2021.101594.
11. Pham, T.H.M.; Zhang, J.; Li, M.; Shen, T.H.; Ko, Y.; Tileli, V.; Luo, W.; Züttel, A. Enhanced Electrocatalytic CO2 Reduction to C2+ Products by Adjusting the Local Reaction Environment with Polymer Binders. *Adv. Energy Mater.* **2022**, *12*, 2103663 (1-10), doi:10.1002/aenm.202103663.
12. Chang, Q.; Lee, J.H.; Liu, Y.; Xie, Z.; Hwang, S.; Marinkovic, N.S.; Park, A.-H.A.; Kattel, S.; Chen, J.G. Electrochemical CO2 Reduction Reaction over Cu Nanoparticles with Tunable Activity and Selectivity Mediated by Functional Groups in Polymeric Binder. *JACS Au* **2022**, *2*, 214–222, doi:10.1021/jacsau.1c00487.
13. Li, J.; Ozden, A.; Wan, M.; Hu, Y.; Li, F.; Wang, Y.; Zamani, R.R.; Ren, D.; Wang, Z.; Xu, Y.; et al. Silica-Copper Catalyst Interfaces Enable Carbon-Carbon Coupling towards Ethylene Electrosynthesis. *Nat. Commun.* **2021**, *12*, 1–10, doi:10.1038/s41467-021-23023-0.
14. Kim, C.; Bui, J.C.; Luo, X.; Cooper, J.K.; Kusoglu, A.; Weber, A.Z.; Bell, A.T. Tailored Catalyst Microenvironments for CO2 Electroreduction to Multicarbon Products on Copper Using Bilayer Ionomer Coatings. *Nat. Energy* **2021**, *6*, 1026–1034, doi:10.1038/s41560-021-00920-8.
15. García de Arquer, F.P.; Dinh, C.T.; Ozden, A.; Wicks, J.; McCallum, C.; Kirmani, A.R.; Nam, D.H.; Gabardo, C.; Seifitokaldani, A.; Wang, X.; et al. CO2 Electrolysis to Multicarbon Products at Activities Greater than 1 A Cm−2. *Science (80-.).* **2020**, *367*, 661–666, doi:10.1126/science.aay4217.
16. Ahn, S.; Klyukin, K.; Wakeham, R.J.; Rudd, J.A.; Lewis, A.R.; Alexander, S.; Carla, F.; Alexandrov, V.; Andreoli, E. Poly-Amide Modified Copper Foam Electrodes for Enhanced Electrochemical Reduction of Carbon Dioxide. *ACS Catal.* **2018**, *8*, 4132–4142, doi:10.1021/acscatal.7b04347.
17. Dutta, A.; Rahaman, M.; Luedi, N.C.; Mohos, M.; Broekmann, P. Morphology Matters: Tuning the Product Distribution of CO2 Electroreduction on Oxide-Derived Cu Foam Catalysts. *ACS Catal.* **2016**, *6*, 3804–3814, doi:10.1021/acscatal.6b00770.
18. Luo, M.; Wang, Z.; Li, Y.C.; Li, J.; Li, F.; Lum, Y.; Nam, D.H.; Chen, B.; Wicks, J.; Xu, A.; et al. Hydroxide Promotes Carbon Dioxide Electroreduction to Ethanol on Copper via Tuning of Adsorbed Hydrogen. *Nat. Commun.* **2019**, *10*, 1–7, doi:10.1038/s41467-019-13833-8.
19. Chen, X.; Chen, J.; Alghoraibi, N.M.; Henckel, D.A.; Zhang, R.; Nwabara, U.O.; Madsen, K.E.; Kenis, P.J.A.; Zimmerman, S.C.; Gewirth, A.A. Electrochemical CO2-to-Ethylene Conversion on Polyamine-Incorporated Cu Electrodes. *Nat. Catal.* **2021**, *4*, 20–27, doi:10.1038/s41929-020-00547-0.
20. Zhuang, T.T.; Liang, Z.Q.; Seifitokaldani, A.; Li, Y.; De Luna, P.; Burdyny, T.; Che, F.; Meng, F.; Min, Y.; Quintero-Bermudez, R.; et al. Steering Post-C-C Coupling Selectivity Enables High Efficiency Electroreduction of Carbon Dioxide to Multi-Carbon Alcohols. *Nat. Catal.* **2018**, *1*, 421–428, doi:10.1038/s41929-018-0084-7.
21. Ma, W.; Xie, S.; Liu, T.; Fan, Q.; Ye, J.; Sun, F.; Jiang, Z.; Zhang, Q.; Cheng, J.; Wang, Y. Electrocatalytic Reduction of CO2 to Ethylene and Ethanol through Hydrogen-Assisted C–C Coupling over Fluorine-Modified Copper. *Nat. Catal.* **2020**, *3*, 478–487, doi:10.1038/s41929-020-0450-0.
22. Marcos-Madrazo, A.; Casado-Coterillo, C.; Irabien, Á. Sustainable Membrane-Coated Electrodes for CO2 Electroreduction to Methanol in Alkaline Media. *ChemElectroChem* **2019**, *6*, 5273–5282, doi:10.1002/celc.201901535.
23. Brée, L.C.; Wessling, M.; Mitsos, A. Modular Modeling of Electrochemical Reactors: Comparison of CO2-Electolyzers. *Comput. Chem. Eng.* **2020**, *139*, 106890, doi:10.1016/j.compchemeng.2020.106890.
24. O'Brien, C.P.; Miao, R.K.; Liu, S.; Xu, Y.; Lee, G.; Robb, A.; Huang, J.E.; Xie, K.; Bertens, K.; Gabardo, C.M.; et al. Single Pass CO2 Conversion Exceeding 85% in the Electrosynthesis of Multicarbon Products via Local CO2 Regeneration. *ACS Energy Lett.* **2021**, *6*, 2952–2959, doi:10.1021/acsenergylett.1c01122.
25. Yano, H.; Tanaka, T.; Nakayama, M.; Ogura, K. Selective Electrochemical Reduction of CO2 to Ethylene at a Three-Phase Interface

on Copper(I) Halide-Confined Cu-Mesh Electrodes in Acidic Solutions of Potassium Halides. *J. Electroanal. Chem.* **2004**, *565*, 287–293, doi:10.1016/j.jelechem.2003.10.021.
